# Namaste Care in nursing care homes for people with advanced dementia: protocol for a feasibility randomised controlled trial

**DOI:** 10.1136/bmjopen-2018-026531

**Published:** 2018-11-25

**Authors:** Katherine Froggatt, Shakil Patel, Guillermo Perez Algorta, Frances Bunn, Girvan Burnside, Joanna Coast, Lesley Dunleavy, Claire Goodman, Ben Hardwick, Julie Kinley, Nancy J Preston, Catherine Walshe

**Affiliations:** 1 International Observatory on End of Life Care, Lancaster University, Lancaster, UK; 2 Department of Health and Human Sciences, University of Herfordshire, Hatfield, UK; 3 Clinical Trials Research Centre, The University of Liverpool, Liverpool, UK; 4 University of Bristol, Bristol, UK; 5 St Chrisptophers Hospice, London, UK

**Keywords:** dementia, namaste care, feasibility study, randomised controlled trial, palliative care, nursing care homes

## Abstract

**Introduction:**

Many people living with advanced dementia live and die in nursing care homes. The quality of life, care and dying experienced by these people is variable. Namaste Care is a multisensory programme of care developed for people with advanced dementia. While there is emerging evidence that Namaste Care may be beneficial for people with dementia, there is a need to conduct a feasibility study to establish the optimum way of delivering this complex intervention and whether benefits can be demonstrated in end-of-life care, for individuals and service delivery. The aim of the study is to ascertain the feasibility of conducting a full trial of the Namaste Care intervention.

**Methods and analysis:**

A feasibility study, comprising a parallel, two-arm, multicentre cluster controlled randomised trial with embedded process and economic evaluation. Nursing care homes (total of eight) who deliver care to those with advanced dementia will be randomly allocated to intervention (delivered at nursing care home level) or control. Three participant groups will be recruited: residents with advanced dementia, informal carers of a participating resident and nursing care home staff. Data will be collected for 6 months. Feasibility objectives concern the recruitment and sampling of nursing homes, residents, informal carers and staff; the selection and timing of primary (quality of dying and quality of life) and secondary clinical outcome measures (person centredness, symptom presence, agitation, quality of life, resource use and costs and residents’ activity monitored using actigraphy). Acceptability, fidelity and sustainability of the intervention will be assessed using semistructured interviews with staff and informal carers.

**Ethics and dissemination:**

This protocol has been approved by NHS Wales Research Ethics Committee 5 (ref: 17/WA0378). Dissemination plans include working with a public involvement panel, through a website (http://www.namastetrial.org.uk), social media, academic and practice conferences and via peer reviewed publications.

**Trial registration number:**

ISRCTN14948133; Pre-results.

**Strengths and limitations of this study**

- Intervention trialled is based on a theoretical model of how the intervention works, drawn from current evidence base and consultation with care home staff, family and experts.
- Public and Patient Involvement (PPI) will greatly inform the ongoing development of the research design and delivery and assist in recruitment, analysis and dissemination.
- Both proxy and objective measures will be measured with this hard to research population.
- Blinding is not possible due to the nature of the intervention.
- The study will not provide data on the effectiveness of the intervention, but will indicate if a further trial to establish effectiveness is feasible.

## Introduction

### Background

Dementia is a life-limiting condition, with a median survival, decreasing with age, of 6.7–1.9 years.^1^ In advanced dementia, an individual requires full assistance with care is chair or bedbound, doubly incontinent and no longer able to communicate verbally (Functional Assessment of Staging of Alzheimer’s Disease (FAST) scale 6–7).^2^ People with dementia often experience a poor quality of death, preceded by a period of poor quality of life, with over and under treatment occurring.^3–5^ There is an increasing urgency for appropriate care that will ensure a good quality of life and dying are achieved.^5 6^

Evidence for therapeutic healthcare interventions for people with advanced dementia is limited. Reviews of therapies such as music therapy indicate mixed outcomes for people with dementia, with a Cochrane review identifying equivocal evidence.^7^ More recent reviews of therapeutic interventions have identified large positive effects on behavioural, cognitive and physiological outcomes,^8^ to moderate effects on anxiety with small effects on behavioural symptoms and evidence for short-term improvement in mood and reduction in behavioural disturbance.^9 10^ In a Cochrane review of touch therapies, some evidence of an effect was identified, but not specifically for people with advanced dementia.^11^ A recent review indicated that massage reduced levels of agitation.^12^ Interventions supporting person-centred care have been shown to reduce agitation and behavioural disturbance. There is some evidence for individualised interventions, within a bio-psychosocial framework, improving behavioural symptoms.^13–15^

Interventions with a single focus on reducing pain, physical symptoms or specific behavioural disturbances have been found to be effective.^3^ It is recognised that for people with advanced dementia there is a need for interventions that complement and enhance pharmacological interventions. This study addresses the lack of evidence available through completed research, to consider the stage-specific efficacy of non-pharmacological interventions.^16^ There is also a need for practical interventions that staff can learn to deliver which allow them to provide person-centred care.

Palliative and end-of-life care interventions for people with dementia that emphasise a person-centred philosophy, and use co-design approaches, are being developed and tested.^17^ Namaste Care is one such intervention. Non-randomised research studies have identified that Namaste Care at the end of life reduces the severity of behavioural and physical symptoms and occupational disruptiveness and may have an impact on social interaction, delirium and agitation.^18–22^ The potential for cost savings with respect to reduced psychotropic medication use has also been indicated.^19 23^ Qualitative evidence suggests greater family and staff satisfaction with care.^18^ However, none of these studies have compared this intervention with other approaches to palliative and end-of-life care for this population. We do not yet know the optimum way of delivering this complex intervention and which benefits (including cost-effectiveness) can be demonstrated in end-of-life care, for individuals and service delivery.

In Phase i of this study, a realist review of 85 papers that considered Namaste Care and sensory interventions (such as music therapy or massage) for people with advanced dementia identified three context–mechanism–outcome configurations. This indicated what needs to be in place for Namaste Care to work for this population. The overarching theme was the importance of providing activities that enabled the development of moments of connection for people with advanced dementia. This can occur when the following three elements are in place: provision of structured access to stimulation (social and physical), equipping care home staff to be able to cope with complex variable behaviours and providing a framework for person-centred care.

### Intervention development

The Namaste Care intervention is already promoted using existing resources.^24 25^ In this study, a four-stage approach to the development and refinement of the intervention resources was used. This entailed (1) collating the existing intervention materials and the findings of the realist review to draft an intervention description; (2) exploring the readability, comprehensibility and utility of the materials with staff unfamiliar with Namaste Care; (3) using a modified nominal group techniques with people with Namaste Care experience to refine and prioritise the intervention implementation materials; and (4) final refinement with the study’s patient and public involvement panel. This led to production of a 16-page A4 booklet. The booklet included the use of flow charts, graphics and colour-coded information supported by infographics, and a training package.

Therefore, we propose undertaking a feasibility cluster controlled randomised trial in a nursing care home context between 1 January 2018 to 31 March 2019.

### Aims and objectives

The primary objective of this feasibility study is to ascertain the feasibility of conducting a full trial of the Namaste Care intervention.

The feasibility issues associated with the research design and data collection processes to enable the design of a full trial to determine the efficacy of Namaste Care are

- To understand how best to sample and recruit nursing homes into a cluster randomised controlled trial of Namaste Care.
- To determine the most appropriate selection, timing and administration of primary and secondary outcome measures for a full cluster randomised controlled trial of Namaste Care against criteria of bias minimisation, burden and acceptability.
- To establish recruitment, retention and attrition rates at the level of the nursing home and individual resident, informal carer and nursing home staff.
- To establish the willingness of a large number of nursing homes representing the range of nursing homes, with respect to provider type, size, resident care needs, to participate in a full trial.
- To assess the acceptability, fidelity and sustainability of the Namaste Care intervention.

Secondary objectives include resident levels of sleep/activity, neuropsychiatric symptoms and pain, informal carer satisfaction with care at the end-of-life staff care giving experiences and satisfaction with care in end-of-life care. Health economic and healthcare resource use will also be assessed.

## Methods and analysis

### Trial design

A feasibility study consisting of a parallel, two-arm, multicentre cluster controlled randomised trial design with an embedded process evaluation is to be conducted. The clustering will take place at the nursing care home level. The Namaste Care programme in the intervention arm will be compared with the standard programme of care used in the control homes.

### Study population

#### Nursing care homes

Eight nursing care homes based in the northwest of England already using a recognised palliative care programme (eg, Gold Standards Framework for Care Homes, Six Steps to Success or equivalent) will be recruited into the study. Two nursing care homes will be allocated to the control arm while six nursing care homes will be allocated to the intervention arm. To meet the eligibility criteria, the nursing care home needs to have

1. At least 30 beds.
2. Six residents who meet the resident eligibility criteria.
3. The space to run the Namaste Care programme.
4. A manager or a nominated person to act as the principal investigator.

A nursing care home will not be eligible to join the study if they

1. Are rated as needs improvement or inadequate in the latest Care Quality Commission (CQC) inspection.
2. Are subject to (CQC)enforcement notices.
3. Have already introduced Namaste Care in their nursing care home.
4. Are currently involved in another research study that conflicts with this study.

#### Individual participants

Residents: To meet the resident eligibility criteria, a resident has to

1. Be a permanent resident living in the participating nursing care home.
2. Lack mental capacity.
3. Have a formal assessment of advanced dementia based on the FAST score of 6–7 made by the nursing care home manager or another experienced member of staff.
4. Have a key worker member of staff willing to complete outcome tools.

A resident will be ineligible to participate in the study if the resident

1. Is permanently bedbound.
2. Is currently or has recently been involved in another research study that conflicts with Namaste Care or with data collection during the course of the Namaste Care study.

Informal carer: To meet the informal carer eligibility criteria, a person who

1. Is 18 years and over.
2. Can communicate in English.
3. Self-defines as a relative or a friend and acts a carer for a resident enrolled to take part in the study.

A person will not be eligible to participate in the study if

1. Their relative or friend is a resident and has not been enrolled in to the study.

Nursing care home staff: To meet the nursing care home eligibility criteria, a person has to be

1. A member of health and social care staff paid to provide care to residents with advanced dementia within participating nursing care homes.

Nursing care home staff will not be eligible to participate in the study if

1. They are in the intervention arm and they have delivered the Namaste Care programme or cared for residents receiving Namaste Care in a nursing care home not involved in this study.

#### Sample size and selection

As the aim of this study is to establish feasibility of a full trial, a formal sample size calculation was not carried out. A sample size of eight nursing homes (six interventions and two controls) has been selected as it offers a reasonable test of the intervention to assess the feasibility objectives. There have been a range in the sample sizes used in feasibility studies in nursing homes ranging from 2^26^6 to 14.^27^

Eligible nursing care homes will be identified through online resources such as the ENRICH database. Following the initial identification, contact will be made with managers of the nursing care home to discuss the study and confirm the eligibility of the nursing care home. Consent for the homes will be assumed when the manager of the facility signs a contract drawn up by the sponsor, Lancaster University.

### Randomisation

The randomisation of participating nursing care homes to either the intervention arm or the control arm will be undertaken by statisticians from the Clinical Trials Research Centre (CTRC) at the University of Liverpool randomisation team who will not be involved in the study. Due to the clustered randomisation approach of this study, all study participants will be assigned to the same study arm as the nursing care home they are associated with. The nature of the intervention and its delivery means that it will not be possible to blind nursing homes or staff to the allocation status. If possible, to minimise potential for bias, staff involved in the delivery of the Namaste Care intervention will not be involved in the completion of outcome measures. It will not be possible to blind researchers to the allocation of nursing homes as the intervention requires changes to the nursing home environment which may be visible to any researcher visiting the facility.

**Figure 1:**
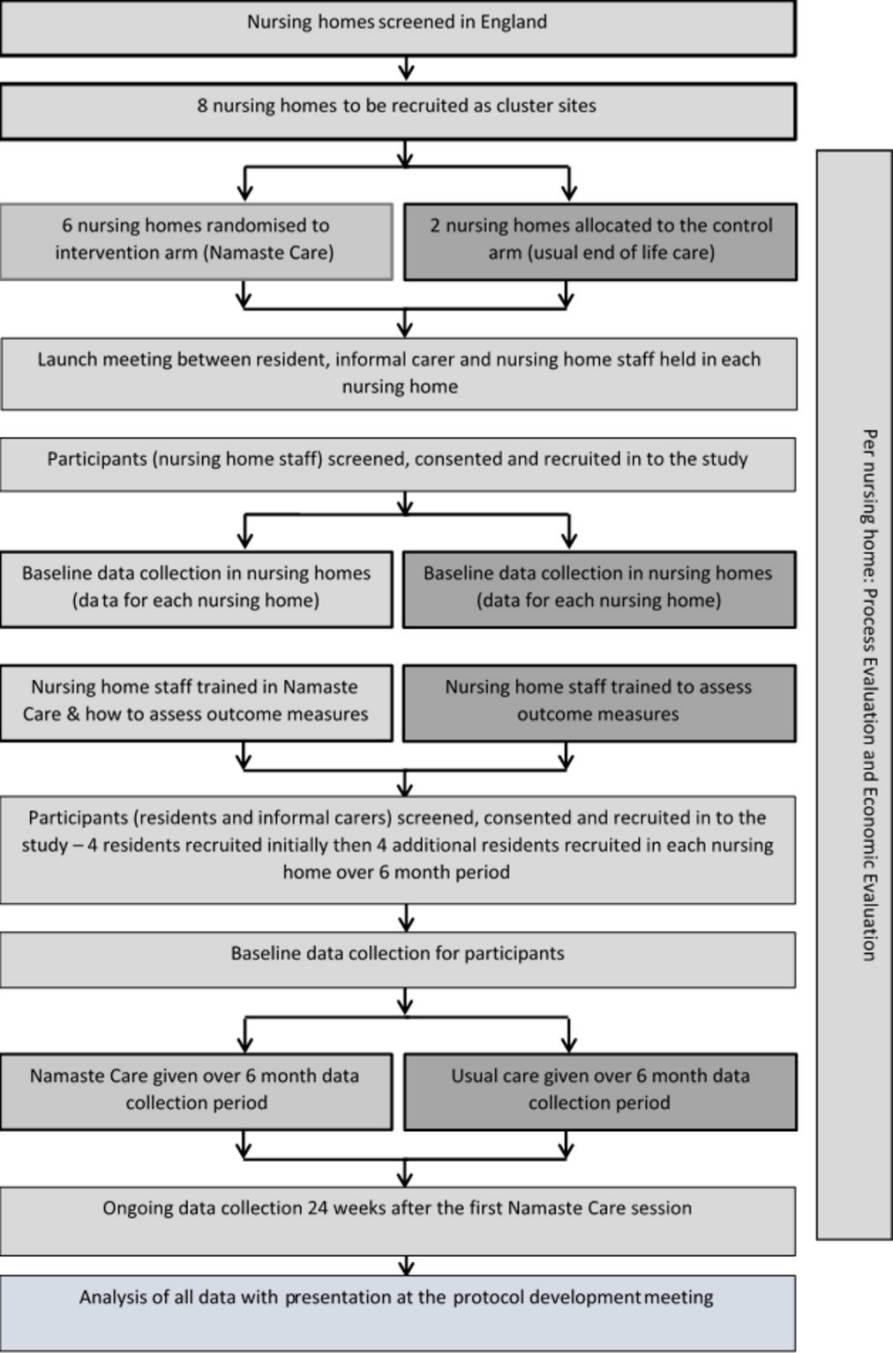
Flow diagram outlining the process of the study.

The study flow chart of activities (figure 1) show the recruitment process to be followed.

### Consent procedures

#### Individual participants

Residents: Potentially eligible residents will be screened by the principal investigator and the senior care team at each nursing care home. Consent for the eligible residents will be sought from a personal consultee of the resident in the first instance. If a person consultee does not reply within month of been given the invitation pack, then assent will be taken from either a nominated consultee or the process used by the nursing care home in question. Once permission is granted by the personal consultee, members of the research team will discuss the study with the personal consultee and gain assent for residents to take part in the study. Process consent will also be considered for the resident participant group.^28^ Therefore, if a resident shows signs of not wanting to take part in the Namaste Care session they will be allowed to miss the Namaste Care session and still continue in the trial.

Informal carer: The informal carers of residents enrolled to the study will be identified by the principal investigator and the senior care team at each nursing care home and invited to consent to complete questionnaires and participate in a qualitative interview.

Nursing care home staff: Nursing care home staff interested in taking part in the Namaste Care study will be identified by the nursing care home manager. On identification, researchers will discuss the study with the identified staff members and obtain written consent from each staff member.

A research lead will be appointed in each nursing care home. The research lead will be tasked with ensuring the paperwork associated with clinical research and the Investigator Site File is maintained. The research lead and the principal investigator from the intervention sites and the control sites will be invited to a training day for guidance on selection of participants and completion of data collection forms and maintaining the Investigator Site File.

Participants will be followed for 6 months after the commencement of the Namaste Care intervention in each nursing care home in the intervention arm or after the recruitment of the first four residents in the nursing care home for sites in the control arm.

### Intervention

The intervention is a programme of care (Namaste Care), delivered in the intervention care homes by care staff working in the facility. The following description uses the TIDieR guidelines for intervention description (items 1–9).^29^

Namaste Care seeks to give comfort and pleasure to people with advanced dementia through engagement, meaningful and creative activities as well as sensory stimulation to reflect the resident’s ‘life story’.^24^ Supporting resource materials have been developed which provide the following guidance regarding the implementation of Namaste Care programme.

- The Namaste Care sessions should be undertaken within a designated space in the nursing home. This space could be within another room or a room which is used for other purposes.
- The environment of the designated space must be made ‘special’ and should enable a feeling of calm that is welcoming and homely, with natural or slightly dimmed lighting, perhaps attractive scents, such as lavender from an aromatherapy diffuser, and with soft music playing.
- The Namaste Care sessions should be undertaken in a group setting.
- Food and drink should be offered to the residents.
- A minimum of two nursing home staff members or volunteers should be present to run the Namaste Care sessions.
- The duration and frequency of Namaste Care delivery as proposed by its originator (2 hours a day, twice a day, 7 days a week) will be promoted.^24^

Namaste Care champions will be appointed in each nursing care home in the intervention arm. At least two care staff (registered nurses, care assistants or activity coordinators) will attend a 1-day workshop about Namaste Care, led by an experienced external facilitator. A follow-up training session will be held at each nursing care home to train more staff and provide advice on preparing the Namaste space.

Prior to the commencement of enrolment, Namaste Care champions (eligible nursing care homes will be identified in the intervention arm) will be appointed in each nursing care home. The Namaste Care champion will be invited to a training day for guidance on Namaste Care intervention, held at a site away from nursing care homes and undertaken by members of the research team and an external trainer. A follow-up training session will be held at each nursing care home.

### Control arm

The care home manager of nursing care homes allocated to the control arm will be asked to continue delivering the usual care programme used in their facility.

Training on the Namaste Care programme will be available to the nursing care homes in the control arm after the study has been completed.

### Outcome and study measures

We consider two contender primary outcomes for a full trial: (1) quality of dying (dementia) (CAD-EOLD) and (2) quality of life (QUALID) (tables 1-4).^30 31^

**Table 1:** Summary of resident data collected by care home staff, outcome measures and time schedule

Data collected and tool used		Pre-intervention	Monthly	At 6 months or death	
Socio-demographics	Age, gender, ethnicity, existing medical conditions, stage of dementia on Functional Assessment of Staging of Alzheimer’s Disease score	x	x	x	
Quality of dying	Measure to assess quality of death using CAD-EOLD (Comfort Assessment in Dying with Dementia)^47 48^	x	x	x	
Quality of life of the person with dementia	EQ-5D-5L^38^ self-rated health index and visual analogue scale of current health state	x	x	x	
Neuropsychiatric Inventory (Neuropsychiatric Inventory–Questionnaire)	Measure to assess psychiatric state of resident using NPI-Q^39^	x	x	x	
Pain	Measure to assess level of pain using PAIN-AD^35^	x	x	x	
Quality of life	EQ-5D-5L	x	x	x	
ICECAP Supportive Care Measure	Health economic measure using ICEPCAP-SCM^39^	x	x	x	
ICECAP-O measure (CEpop CAPability measure for Older people)	Health economic measure using ICEPCAP-O^49^	x	x	x	–
Cohen-Mansfield Agitation Inventory	Measure to assess resident agitation^36^	x	x	x	
ICECAP Supportive Care Measure using Think Aloud	Health economic measure using ICEPCAP-SCM using Think Aloud	x	x	x	
ICECAP-O measure using Think Aloud	Health economic measure using ICECAP-O using Think Aloud	x	x	x	

**Table 2:** Summary of informal carer data collected, as assessed by informal carers, outcome measures and time schedule

Data collected and tool used		Baseline	At 1 month	At 6 months or death
Socio-demographics	Age, gender, ethnicity, existing medical conditions	x	–	–
Service use in the prior month	Client Service Receipt Inventory^50^ Calculates service and total care costs	x	x	x
Quality of life of the carer	EQ-5D-5L	x	x	x
Satisfaction with care	SWC-EOLD (Satisfaction with Care at the End-of-Life in Dementia)^34^	x	x	x
ICECAP Close Person Measure of health economic evaluation	Health economic evaluation using ICECAP-CPM	x	x	x
ICECAP Close Person Measure of health economic evaluation	Health economic evaluation using ICEPCAP-CPM completing using Think Aloud	x	x	x

**Table 3:** Summary of staff data collected as assessed by care home staff: outcome measures and time schedule

Data collected and tool used		Preintervention	6 months
Staff socio-demographics	Age, gender, ethnicity	X	–
Staff work characteristics	Highest qualification, role in care home, length of service	X	–
Organisational support for person-centred care	The Person-Centred Care Assessment Tool 32	X	X
Organisational support for readiness for change	The Alberta Context Tool Questionnaire^51^	X	–

**Table 4:** Summary of nursing care home level data collected, outcome measures, time schedule and the type of person assessing the outcome measure

Data collected and tool used		Pre intervention	Monthly	At 6 months only	Postintervention
Care home occupancy level	Number of available beds to new residents	S	–	–	–
Cost of living in the care home	Fees to live in the care home	S	–	–	–
Contributions from local government	Fees paid by the local government for each resident	S	–	–	–
Staffing levels	Number and type of staff	S	–	–	–
Number of GP practices the care home works with	Number of GP practices the care home works with	S	–	–	–
Number of GPs the care home works with	Number of GPs the care home works with	S	–	–	–
Level of need of residents in the care home	Amount of support each resident needs	S	–	–	–
Staff turnover and sickness levels	Number of staff in the care home and monthly sickness record	S	S	–	–
Ambulances and hospital use	Number and length of hospital admissions (days), A&E attendances and readmissions	S	S	S	–
Number of hospital admissions	Respiratory infections, urinary tract infections, dehydration, congestive heart failure?	S	S	S	–
Out of hours GP contacts	GP visits or telephone contact	R	R	R	R

Measure assessed by S: care home staff; R: researcher.

The secondary outcome measures in this trial (table 1) will measure measure person centredness, symptom presence, agitation, quality of life, resource use and costs; and sleep and activity using actigraphy.^32–37^Semistructured interviews with staff and informal carers will assess perceptions of Namaste Care or usual care, assessment of the fidelity, acceptability and appropriateness of Namaste Care or of usual care.

The outcome measures to be used are listed in tables 1–4 and presented based on respondent type, that is, measures for residents (table 1), informal carers (table 2), staff (table 3) and at the level of the nursing care home (table 4). At the start of the study, descriptive data will be collected for all participating nursing care homes such as ownership and funding model, size, staffing, case mix, staff turnover, staff sickness/absence and geographical location. An interview with the nursing care home manager will also be conducted to ascertain the organisation’s readiness for change.

### Data collection

In this study, the outcome measures and process evaluation data will be gathered via five different methods:

1. Questionnaires: The nursing home staff participant group and the informal carer participant group will be asked to complete written questionnaires at timepoints outlined in tables 1–3. The questionnaires for the resident participant group will be proxy completed by nursing care home staff who are key workers for the participating residents. Note the time frame for baseline varies depending on the participant group. Data on nursing home level data about engagement with health and social care services will be collected using standardised data collection forms (table 4).
2. Objective measures: The participating residents will be asked to wear an actigraph watch-like device for 28 days from the baseline visit. This actigraph will be placed on the wrist or ankle of the resident and will be used to continuously measure sleep and activity.
3. Interviews: Semi structured interviews will be undertaken at the baseline with the nursing home manager and at the end of the data collection period with family carers and care staff.
4. Observations of the residents will be undertaken intermittently during the delivery of the care programme and during the delivery of usual care in the control sites.
5. Data logs will be completed in the intervention sites using a proforma to record intervention delivery.

#### Feasibility work for economic evaluation

The use of a number of potential outcome measures will be explored in terms of feasibility and acceptability of proxy completion with the particular population, evaluated through the Think Aloud technique. The chosen measures are included in the National Institute for Health and Care Excellence-recommended measures for health and social care: EQ-5D-5L (five items), the ICECAP-O (five items) and the ICECAP-Supportive Care Measure (ICECAP-SCM) (seven items).^38–40^ A Think Aloud technique will also be used with the ICECAP-O, ICECAP-SCM and ICECAP-CPM tools for a proportion of participants at 2, 4 and 24 weeks, to obtain 20–30 Think Aloud interviews across a range of timepoints.^41^ This Think Aloud technique will be undertaken either via telephone or face to face. The feasibility of collecting resource use data through nursing home records will be assessed, and the cost of the interventions will be estimated, for use in a full evaluation.

#### Process evaluation

The process evaluation elements of the study (table 5) will address staff members’ perceptions of Namaste Care (intervention arm) or perceptions of the effectiveness of usual care (control arm) using interviews approximately 24 weeks after the first resident is recruited at the nursing home. Family carers’ perceptions of Namaste Care (intervention arm) or carers’ perceptions of the effectiveness of usual care (control arm) will be ascertained using interviews between 16–24 weeks after the first resident is recruited at the nursing home.

**Table 5:** Data collected as part of the process evaluation

Outcome measures or rationale for data collection	Data collected through	Time of data collection
To assess carers’ perceptions of Namaste Care (intervention arm) or carers’ perceptions of the effectiveness of usual care (control arm)	Interviews conducted by the researcher	Approximately 16–24 weeks after the first resident is recruited at the nursing home (If a resident dies during the trial then the informal carer will be approached at least 8 weeks after the resident’s death)
Staff members’ perceptions of Namaste Care (intervention arm) or perceptions of the effectiveness of usual care (control arm)	Interviews conducted by the researcher	Approximately 24 weeks after the first resident is recruited at the nursing home
To assess the fidelity, acceptability and appropriateness of Namaste Care (intervention arm) or assess effectiveness of usual care (control arm)	Observations conducted by the researcher	Approximately 2, 4 and 24 weeks after the start of the intervention for nursing homes in the intervention arm Approximately 2 and 4 weeks after the first resident is recruited for nursing homes in the control arm
To assess the fidelity, acceptability and appropriateness of the Namaste Care (intervention arm)	Data log completed by the staff delivering the Namaste Care session	Throughout the intervention

To assess the fidelity, acceptability and appropriateness of Namaste Care (intervention arm) or assess effectiveness of usual care (control arm) observation will be conducted at approximately 2, 4 and 24 weeks after the start of the intervention for nursing homes in the intervention arm and approximately 2 and 4 weeks in the control arm.

A data log will be completed by the staff delivering the Namaste Care session throughout the intervention delivery.

### Data management

Data management is provided by the CTRC at the University of Liverpool. Paper-based case report forms will be written to record data in a consistent way and ensure anonymisation of the data. Data stored at the CTRC will be checked for missing or unusual values (range checks) and checked for consistency within participants over time. Any suspect data will be returned to the site in the form of data queries. Data query forms will be produced at the CTRC from the trial database and sent either electronically or through the post to a named individual (as listed on the site delegation log). Sites will respond to the queries providing an explanation/resolution to the discrepancies and return the data query forms to CTRC. The forms will then be filed along with the appropriate data collection forms and the appropriate corrections made on the database. The process of database lock, unlock and closure will be followed according to the CTRC policy.

### Data analysis plan

Three types of data will be analysed: quantitative date from surveys and the actigraphs, qualitative data from interviews and economic data.

#### Quantitative analysis

Outcomes at baseline and follow-up will be summarised using descriptive statistics and will be used to make a decision on undertaking a full trial. Analysis of the outcome data will focus on recruitment, response and completion rates, and missing data. Reasons for non-consent and missing outcome data will be reported. Estimates of SD and proxy agreement will be determined, and construct validity estimated intracluster correlation coefficient will be made.

The sleep/activity data from the actigraph will be analysed using summary statistics for the sleep analysis data (sleep/wake ratios, total sleep time, sleep efficiency, wake after sleep onset and total activity); participant’s rhythm fragmentation and synchronisation will be estimated via intradaily variability (IV) and interdaily stability (IS).^42 43^ The actigraph will be used to ascertain the feasibility of use this outcome measure to collect data in a full trial.

#### Qualitative analysis

Semistructured interviews will be audio-recorded, transcribed and anonymised. Framework analysis will be used in the analysis of qualitative data, with data collection, management and analysis rigorously conducted to enable reporting against COREQ guidelines. Group/individual interviews and observation sessions will be digitally audio-recorded and fully transcribed. NVivo will be used to facilitate data management and analysis as this supports framework analysis techniques.

#### Analysis of economic data

Economic assessments of relevant outcome measures will combine qualitative assessments of feasibility of use for the outcome measures gained through the think aloud techniques and more quantitative assessments of agreement between proxies, and assessments of construct validity for the measures.^44^ Response and completion rates will be assessed. Constant comparative analytical methods will be used to provide a more in-depth assessment of both the questionnaire completion and respondents’ perceptions of the measures in the Think Aloud interviews.

Unit cost information will be generated using bottom-up costing for the Namaste intervention itself, ensuring that a cost for the intervention will be available in a full trial. Other sources of unit cost information will be identified and collated for use in a future full trial and will be applied to the collected resource use data to enable the preliminary assessment of costs and benefits, and the main cost drivers for a full evaluation. All data will be costed using unit cost data in pounds sterling, and from a single year, as close as possible to the end of the feasibility study.

### Public and patient involvement (PPI)

Two carer representatives from the Alzheimer’s Society Research Network UK were co-applicants as part of the core study/trial management group. They will be present at all project teleconferences and meetings. A Public Involvement Panel will be established in the north west of England. This will comprise of six to eight members, co-chaired by the PPI co-applicants. The members have personal experience of family members living with dementia in care homes. The panel members will work alongside the research team to assist in different areas of research including reviewing participant information sheets and other documentation, five face-to-face meetings are proposed during the study, and communication between meetings will be by regular updates. There will also be PPI representation on the research advisory group and Trial Steering Committee (TSC).

### Monitoring and trial management

For this research population there is a relatively high risk of death, hospitalisation or progression of disease for participants during the course of the study but which are not anticipated to be related to the receipt of the intervention. This level and type of risk will be treated as an acceptable risk for the purposes of the study and will not constitute adverse events or serious adverse events unless concern is raised by anyone associated with the study that these events could be directly related to participation in this study.

The Trial Management Group is responsible for (1) protocol completion, (2) obtaining ethical approval for phases I and II, (3) obtaining ethical approval for phase III plus nursing home approval process; (4) appointing and facilitating the TSC; and (5) working with the dissemination partners. The group will meet for a ‘kick off’ meeting face to face at the start of the project. Thereafter there will be monthly teleconferences and twice yearly face-to-face meetings. The TSC, with an independent chair, will provide overall supervision of the trial including trial progress and participant safety. Membership will be drawn from experts in health services research, nursing home research and PPI. They will meet prior to the start of the trial phase and then twice during the second year of the project. The TSC will have the role of a traditional Data Monitoring Committee as this a feasibility study with a low-risk intervention. A TSC charter based on the guidelines published by the National Institute for Health Research (NIHR) will be used to identify the remit of the TSC. An International Advisory Group will also be established to provide external expert advice on the overall progress of the study. There is a data management plan (held by the sponsor) which outlines data storage periods and future access to data.

## Discussion

This protocol describes the Namaste Care programme for residents with advanced dementia who are living in nursing care homes. The Namaste Care programme is a multisensory care programme conducted on a daily basis in a group setting. This study will provide information on implementation, cost and acceptability of a defined intervention. In addition, this study will provide information on usefulness, practicality and acceptability of the selected outcome measures and processes used in this study. In conclusion, the findings of this study will inform future research on the Namaste Care programme in nursing care homes.

## Ethics and dissemination

The study has been approved by the Wales Research Ethics Committee 5 (ref: 17/WA/0378; V.04. 9 February 2018). As resident’s eligible for the study will lack capacity to consent, consent for residents will be taken from either a personal consultee or a nominated consultee following the Mental Capacity Act (2005) guidance.^28 45^ A procedure for reporting issues of concern in the care setting has been written.

The following dissemination channels will be used: a project website (http://www.namastetrial.org.uk), a leaflet summarising the study, summaries of findings, publications/articles for general as well as scientific media and social media such as Twitter (@NamasteResearch). All publications will follow the relevant reporting guidelines for reviews and trials.^46^

## Supplementary Material

Reviewer comments

Author's manuscript
