# Supplementary material for: Namaste Care in nursing care homes for people with advanced dementia: protocol for a feasibility randomised controlled trial
Source: BMJ Open. 2018 Nov 25;8(11):e026531. doi: 10.1136/bmjopen-2018-026531 (PMC6254402; doi:10.1136/bmjopen-2018-026531)
Supplement: Author's manuscript [file bmjopen-2018-026531.draft_revisions.pdf]

BMJ Open is committed to open peer review. As part of this commitment we make the peer review history of every article we publish publicly available.

When an article is published we post the peer reviewers' comments and the authors' responses online. We also post the versions of the paper that were used during peer review. These are the versions that the peer review comments apply to.

The versions of the paper that follow are the versions that were submitted during the peer review process. They are not the versions of record or the final published versions. They should not be cited or distributed as the published version of this manuscript.

BMJ Open is an open access journal and the full, final, typeset and author-corrected version of record of the manuscript is available on our site with no access controls, subscription charges or pay-per-view fees (http://bmjopen.bmj.com).

If you have any questions on BMJ Open's open peer review process please email editorial.bmjopen@bmj.com

BMJ Open

Namaste Care in nursing care homes with people with advanced dementia: protocol for a feasibility randomised controlled trial

Journal:	*BMJ Open*
Manuscript ID	bmjopen-2018-026531
Article Type:	Protocol
Date Submitted by the Author:	18-Sep-2018

Complete List of Authors:	Froggatt, Katherine ; Lancaster University, Faculty of Health and Medicine<br>Patel, Shakil; Lancaster University Faculty of Health and Medicine<br>Perez Algorta, Guillermo; Lancaster University Faculty of Health and Medicine<br>Bunn, Frances; University of Herfordshire, Department of Health and Human Sciences<br>Burnside, Girvan; University of Liverpool<br>Coast, Joanna; University of Bristol<br>Dunleavy, Lesley; Lancaster University Faculty of Health and Medicine<br>Goodman, Claire; University of Hertfordshire, Centre for Research in Primary and Community Care<br>Hardwick, Ben; University of Liverpool<br>Kinley, Julie; St Chrisptophers Hospice<br>Preston, Nancy J.; Univ Lancaster<br>Walshe, Catherine; Lancaster University

Keywords:	Dementia < NEUROLOGY, Namaste Care, Feasibility study, Randomised controlled trial, PALLIATIVE CARE, Nursing care homes

SCHOLARONE™
Manuscripts

Namaste Care in nursing care homes with people with advanced dementia: protocol for a feasibility randomised controlled trial

Katherine Froggatt[1], Shakil Patel[1], Guillermo Perez Algorta[1], Frances Bunn[2], Girvan Burnside[4], Joanna Coast[3], Lesley Dunleavy[1], Claire Goodman[2], Ben Hardwick[4], Julie Kinley[5], Nancy Preston[1], Catherine Walshe[1].

1. Lancaster University. 2. University of Hertfordshire. 3. University of Bristol. 4. The University of Liverpool. 5. St. Christopher's Hospice.

Froggatt, Katherine k.froggatt@lancaster.ac.uk; Patel, Shakil s.patel16@lancaster.ac.uk; Perez Algorta, Guillermo g.perezalgorta@lancaster.ac.uk; Frances Bunn f.bunn@herts.ac.uk; Girvan Burnside g.burnside@liverpool.ac.uk; Jo Coast jo.coast@bristol.ac.uk; Dunleavy, Lesley l.dunleavy@lancaster.ac.uk; Ben Hardwick b.hardwick1@liverpool.ac.uk; Julie Kinley julie@kinley.me.uk; Preston, Nancy n.j.preston@lancaster.ac.uk; Walshe, Catherine c.walshe@lancaster.ac.uk

Corresponding author

Katherine Froggatt

Word Count – 4600 without references

For peer review only - http://bmjopen.bmj.com/site/about/guidelines.xhtml

ABSTRACT (300 words –307)

Introduction:

Many people living with advanced dementia live and die in nursing care homes. The quality of life, care and dying experienced by these people is variable. Namaste Care is a multi-sensory programme of care that has been developed to provide care for people with advanced dementia. Whilst there is emerging evidence that Namaste Care may be beneficial for people with dementia, there is a need to conduct a feasibility study to establish the optimum way of delivering this complex intervention and whether benefits can be demonstrated in end of life care, for individuals and service delivery.

Methods and analysis:

A feasibility study, comprising a parallel, two-arm, multi-centre cluster controlled randomised trial with embedded process and economic evaluation. Nursing care homes (total of 8) who deliver care to those with advanced dementia will be randomly allocated to intervention (delivered at nursing care home level) or control. Three participant groups will be recruited: residents with advanced dementia; informal carers of a participating resident and nursing care home staff. Data will be collected for 6 months. Primary outcome measures: quality of dying (dementia) and quality of life, Secondary outcome measures will measure person centeredness, symptom presence, agitation, quality of life, resource use and costs. Residents' activity will be monitored using actigraphy. Semi-structured interviews with staff and informal carers will assess perceptions of Namaste Care or effectiveness of usual care, assessment of the fidelity, acceptability and appropriateness of Namaste Care or of usual care.

Ethics and dissemination: This protocol has been approved by NHS Wales Research Ethics Committee 5 (Ref: 17/WA0378). Dissemination plans include working with a public involvement panel, through a website (wwww.namastetrial.org.uk), social media, academic and practice conferences and via peer reviewed publications.

Registration: ISRCTN14948133

For peer review only

Article Summary

Strengths and Limitations of this study

- PPI will greatly inform the ongoing development of the research design and delivery and assist in recruitment, analysis and dissemination
- The intervention trialled was based on a theoretical model of how the intervention works, drawn from the current evidence base, and then in consultation with care home staff, family and experts further revised.
- The study will not provide data on the effectiveness of the intervention, but will indicate if a further study is either warranted and or feasible

For peer review only

For peer review only - http://bmjopen.bmj.com/site/about/guidelines.xhtml

INTRODUCTION

Background

Dementia is a life limiting condition, with a median survival, decreasing with age, of 6.7 to 1.9 years.[1] In advanced dementia, an individual requires full assistance with care, is chair or bedbound, doubly incontinent and no longer able to communicate verbally (FAST scale 6-7).[2] People with dementia often experience a poor quality of death, preceded by a period of poor quality of life, with over and under treatment occurring.[3-5] There is an increasing urgency for appropriate care that will ensure a good quality of life and dying are achieved.[5, 6]

Evidence for therapeutic healthcare interventions for people with advanced dementia is limited. Reviews of therapies such as music therapy indicate mixed outcomes for people with dementia, with a Cochrane review identifying equivocal evidence.[7] More recent reviews of therapeutic interventions have identified large positive effects on behavioural, cognitive and physiological outcomes,[8] to moderate effects on anxiety with small effects on behavioural symptoms and evidence for short term improvement in mood and reduction in behavioural disturbance.[9, 10] In a Cochrane review of touch therapies, some evidence of an effect was identified, but not specifically for people with advanced dementia.[11] A recent review indicated that massage reduced levels of agitation.[12] Interventions supporting person-centred care have been shown to reduce agitation and behavioural disturbance. There is some evidence for individualised interventions, within a bio-psychosocial framework, improving behavioural symptoms.[13-15]

Interventions with a single focus on reducing pain, physical symptoms or specific behavioural disturbances have been found to be effective.[3] It is recognised that for people with advanced dementia there is a need for interventions that complement and enhance pharmacological interventions. This study addresses the lack of evidence available through completed research, to consider the stage specific efficacy of non-pharmacological interventions.[16] There is also a need for practical interventions that staff can learn to deliver which allow them to provide person-centred care.

Palliative and end of life care interventions for people with dementia that emphasise a person-centred philosophy, and use co-design approaches, are being developed and tested.[17] Namaste Care is one such intervention. Non-randomised research studies have identified that Namaste Care at the end of life reduces the severity of behavioural and physical symptoms and occupational disruptiveness and may have an impact on social interaction, delirium and agitation.[18-22] The potential for cost savings with respect to reduced psychotropic medication use has also been

For peer review only - http://bmjopen.bmj.com/site/about/guidelines.xhtml

indicated.[19, 23] Qualitative evidence suggests greater family and staff satisfaction with care.[18] However, none of these studies have compared this intervention with other approaches to palliative and end of life care for this population. We do not yet know the optimum way of delivering this complex intervention and which benefits (including cost-effectiveness) can be demonstrated in end of life care, for individuals and service delivery.

In Phase 1 of this study, a realist review of 85 papers that considered Namaste Care and sensory interventions (such as music therapy or massage) for people with advanced dementia identified three context-mechanism-outcome configurations. This indicated what needs to be in place for Namaste Care to work for this population. The overarching theme was the importance of providing activities that enabled the development of moments of connection for people with advanced dementia. This can occur when the following three elements are in place: provision of structured access to stimulation (social and physical), equipping care home staff to be able to cope with complex variable behaviours, and providing a framework for person-centred care.

Intervention Development

The Namaste Care intervention is already promoted using existing resources. [24, 25] In this study, a four-stage approach to the development and refinement of the intervention resources was used. This entailed: 1. Collating the existing intervention materials and the findings of the realist review to draft an intervention description; 2. Exploring the readability, comprehensibility and utility of the materials with staff unfamiliar with Namaste Care; 3. Using a modified nominal group techniques with people with Namaste Care experience to refine and prioritise the intervention implementation materials; and 4. Final refinement with the study's patient and public involvement panel. This led to production of a 16 page A4 booklet. The booklet included the use of flow charts, graphics and colour coded information supported by infographics, and a training package.

Therefore, we propose undertaking a feasibility cluster controlled randomized trial in a nursing care home context between 01 Jan 2018 to 31 March 2019.

Aims and Objectives

The primary objective of this feasibility study is to ascertain the feasibility of conducting a full trial of the Namaste Care intervention.

The feasibility issues associated with the research design and data collection processes to enable the design of a full trial to determine the efficacy of Namaste Care are:

For peer review only - http://bmjopen.bmj.com/site/about/guidelines.xhtml

a) To understand how best to sample and recruit nursing homes into a cluster randomised controlled trial of Namaste Care;
b) To determine the most appropriate selection, timing and administration of primary and secondary outcome measures for a full cluster randomised controlled trial of Namaste Care against criteria of bias minimization, burden, and acceptability;
c) To establish recruitment, retention and attrition rates at the level of the nursing home and individual resident, informal carer and nursing home staff;
d) To establish the willingness of a large number of nursing homes representing the range of nursing homes, with respect to provider type, size, resident care needs, to participate in a full trial;
e) To assess the acceptability, fidelity and sustainability of the Namaste Care intervention.

Secondary objectives include resident levels of sleep/activity, neuropsychiatric symptoms and pain, informal carer satisfaction with care at the end of life staff care giving experiences and satisfaction with care in end of life care. Health economic and healthcare resource use will also be assessed.

METHODS AND ANALYSIS

Trial Design

A feasibility study consisting of a parallel, two-arm, multi-centre cluster controlled randomised trial design with an embedded process evaluation is to be conducted. The clustering will take place at the nursing care home level. The Namaste Care programme in the intervention arm will be compared with the standard programme of care used in the control homes.

Study Population

Nursing Care Homes

Eight nursing care homes based in the North West of England already using a recognised palliative care programme (for example, Gold Standards Framework for Care Homes, Six Steps to Success or equivalent) will be recruited into the study. Two nursing care homes will be allocated to the control arm whilst six nursing care homes will be allocated to the intervention arm. To meet the eligibility criteria, the nursing care home needs to have:

1. at least 30 beds

For peer review only - http://bmjopen.bmj.com/site/about/guidelines.xhtml

2. 6 residents who meet the resident eligibility criteria
3. the space to run the Namaste Care programme
4. a manager or a nominated person to act as the Principal Investigator.

A nursing care home will not be eligible to join the study if they:

1. are rated as Needs Improvement or Inadequate in the latest CQC inspection
2. are subject to CQC enforcement notices
3. have already introduced Namaste Care in their nursing care home
4. are currently involved in another research study that conflicts with this study.

Individual Participants

Residents – To meet the resident eligibility criteria, a resident has to:

1. be a permanent resident living in the participating nursing care home
2. lack mental capacity
3. have a formal assessment of advanced dementia based on the Functional Assessment of Staging of Alzheimer's Disease (FAST) score of 6-7 made by the nursing care home manager or another experienced member of staff
4. have a key worker member of staff willing to complete outcome tools.

A resident will be ineligible to participate in the study if the resident:

1. is permanently bedbound
2. is currently or has recently been involved in another research study that conflicts with Namaste Care or with data collection during the course of the Namaste Care study.

Informal carer – To meet the informal carer eligibility criteria, a person who:

1. is 18 years and over
2. can communicate in English
3. self-defines as a relative or a friend and acts a carer for a resident enrolled to take part in the study.

A person will not be eligible to participate in the study if:

For peer review only - http://bmjopen.bmj.com/site/about/guidelines.xhtml

1. their relative or friend is a resident and has not been enrolled in to the study.

Nursing care home staff – To meet the nursing care home eligibility criteria, a person has to be

1. a member of health and social care staff paid to provide care to residents with advanced dementia within participating nursing care homes.

Nursing care home staff will not be eligible to participate in the study if

1. they are in the intervention arm and they have delivered the Namaste Care programme or cared for residents receiving Namaste Care in a nursing care home not involved in this study.

Sample Size and Selection

As the aim of this study is to establish feasibility of a full trial, a formal sample size calculation was not carried out. A sample size of 8 nursing homes (6 intervention and 2 controls) has been selected as it offers a reasonable test of the intervention to assess the feasibility objectives. There have been a range in the sample sizes used in feasibility studies in nursing homes ranging from 2,[26] 6 to 14.[27]

Eligible nursing care homes will be identified through online resources such as the ENRICH database. Following the initial identification, contact will be made with managers of the nursing care home to discuss the study and confirm the eligibility of the nursing care home. Consent for the homes will be assumed when the manager of the facility signs a contract drawn up by the Sponsor, Lancaster University.

Randomisation

The randomisation of participating nursing care homes to either the intervention arm or the control arm will be undertaken by statisticians from the Clinical Trials Research Centre (CTRC) at the University of Liverpool randomisation team who will not be involved in the study. Due to the clustered randomisation approach of this study, all study participants will be assigned to the same study arm as the nursing care home they are associated with. The nature of the intervention and its delivery means that it will not be possible to blind nursing homes or staff to the allocation status. If possible, to minimise potential for bias, staff involved in the delivery of the Namaste Care intervention will not be involved in the completion of outcome measures. It will not be possible to

For peer review only - http://bmjopen.bmj.com/site/about/guidelines.xhtml

blind researchers to the allocation of nursing homes, as the intervention requires changes to the nursing home environment which may be visible to any researcher visiting the facility.

The study flow chart of activities (Figure 1) shows the recruitment process to be followed.

Consent Procedures

Individual Participants

Residents – Potentially eligible residents will be screened by the Principal Investigator and the senior care team at each nursing care home. Consent for the eligible residents will be sought from a personal consultee of the resident in the first instance. If a person consultee does not reply within month of been given the invitation pack then assent will be taken from either a nominated consultee or the process used by the nursing care home in question. Once permission is granted by the personal consultee, members of the research team will discuss the study with the personal consultee and gain assent for residents to take part in the study. Process consent will also be considered for the resident participant group.[28] Therefore, if a resident shows signs of not wanting to take part in the Namaste Care session they will be allowed to miss the Namaste Care session and still continue in the trial.

Informal carer – The informal carers of residents enrolled to the study will be identified by the Principal Investigator and the senior care team at each nursing care home and invited to consent to complete questionnaires and participate in a qualitative interview.

Nursing care home staff – Nursing care home staff interested in taking part in the Namaste Care study will be identified by the nursing care home manager. Upon identification, researchers will discuss the study with the identified staff members and obtain written consent from each staff member.

A research lead will be appointed in each nursing care home. The research lead will be tasked with ensuring the paperwork associated with clinical research and the Investigator Site File is maintained. The research lead, and the Principal Investigator from the intervention sites and the control sites will be invited to a training day for guidance on selection of participants and completion of data collection forms and maintaining the Investigator Site File.

Participants will be followed for six months after the commencement of the Namaste Care intervention in each nursing care home in the intervention arm or after the recruitment of the first four residents in the nursing care home for sites in the control arm.

For peer review only - http://bmjopen.bmj.com/site/about/guidelines.xhtml

Intervention

The intervention is a programme of care (Namaste Care), delivered in the intervention care homes by care staff working in the facility. The following description uses the TIDieR guidelines for intervention description (items 1-9).[29]

Namaste Care seeks to give comfort and pleasure to people with advanced dementia through engagement, meaningful and creative activities as well as sensory stimulation to reflect the resident's 'life story'[24]. Supporting resource materials have been developed which provide the following guidance regarding the implementation of Namaste Care programme.

- The Namaste Care sessions should be undertaken within a designated space in the nursing home. This space could be within another room, or a room which is used for other purposes
- The environment of the designated space must be made 'special' and should enable a feeling of calm i.e. welcoming and homely, with natural or slightly dimmed lighting, perhaps attractive scents, such as lavender from an aromatherapy diffuser, and with soft music playing
- The Namaste Care sessions should be undertaken in a group setting
- Food and drink should be offered to the residents
- A minimum of two nursing home staff members or volunteers should be present to run the Namaste Care sessions
- The duration and frequency of Namaste Care delivery as proposed by its originator (two hours a day, twice a day, seven days a week) will be promoted.[24]

Namaste Care champions will be appointed in each nursing care home in the intervention arm. At least two care staff (registered nurses, care assistants or activity coordinators) will attend a one day workshop about Namaste Care, led by an experienced external facilitator. A follow up training session will be held at each nursing care home to train more staff and provide advice on preparing the Namaste space.

Prior to the commencement of enrolment, Namaste Care champions (Eligible nursing care homes will be identified in the intervention arm) will be appointed in each nursing care home. The Namaste Care champion will be invited to a training day for guidance on Namaste Care intervention, held at a site away from nursing care homes and undertaken by members of the research team and an external trainer. A follow up training session will be held at each nursing care home.

For peer review only - http://bmjopen.bmj.com/site/about/guidelines.xhtml

Control Arm

The care home manager of nursing care homes allocated to the control arm will be asked to continue delivering the usual care programme used in their facility.

Training on the Namaste Care programme will be available to the nursing care homes in the control arm after the study has been completed.

Outcome and Study Measures

We consider two contender primary outcomes for a full trial: (1) quality of dying (dementia) (CAD-EOLD) and (2) quality of life (QUALID) (Table 1 -4). [30, 31]

The secondary outcome measures in this trial (Table 1) will measure: measure person-centeredness, symptom presence, agitation, quality of life, resource use and costs; and sleep and activity using actigraphy.[32-37] Semi-structured interviews with staff and informal carers will assess perceptions of Namaste Care or usual care, assessment of the fidelity, acceptability and appropriateness of Namaste Care or of usual care.

The outcome measures to be used are listed in Tables 1 – 4 and presented based on respondent type i.e. measures for residents (Table 1), informal carers (Table 2), staff (Table 3) and at the level of the nursing care home (Table 4). At the start of the study, descriptive data will be collected for all participating nursing care homes such as ownership and funding model, size, staffing, case mix, staff turnover, staff sickness/absence and geographical location. An interview with the nursing care home manager will also be conducted to ascertain the organisation's readiness for change.

Data Collection

In this study, the outcome measures and process evaluation data will be gathered via 5 different methods:

1) Questionnaires – The nursing home staff participant group and the informal carer participant group will be asked to complete written questionnaires at timepoints outlined in Tables 1 – 3. The questionnaires for the resident participant group will be proxy completed by nursing care home staff who are key workers for the participating residents. Note, the timeframe for baseline varies depending on the participant group. Data on nursing home level data about engagement with health and social care services will be collected using standardised data collection forms (Table 4)

For peer review only - http://bmjopen.bmj.com/site/about/guidelines.xhtml

2) Objective measures – The participating residents will be asked to wear an actigraph watch-like device for 28 days from the baseline visit. This actigraph will be placed on the wrist or ankle of the resident and will be used to continuously measure sleep and activity
3) Interviews - Semi-structured interviews will be undertaken at the baseline with the nursing home manager and at the end of the data collection period with family carers and care staff
4) Observations of the residents will be undertaken intermittently during the delivery of the care programme and during the delivery of usual care in the control sites
5) Data logs will be completed in the intervention sites using a proforma to record intervention delivery.

Feasibility Work for Economic Evaluation

The use of a number of potential outcome measures will be explored in terms of feasibility and acceptability of proxy completion with the particular population, evaluated through the think aloud technique. The chosen measures are included in the NICE recommended measures for health and social care: EQ-5D-5L (5 items), the ICECAP-O (5 items) and the ICECAP-Supportive Care Measure (ICECAP-SCM) (7 items).[38-40] A think aloud technique will also be used with the ICECAP-O, ICECAP-SCM and ICECAP-CPM tools for a proportion of participants at 2 weeks, 4 weeks and 24 weeks, to obtain 20-30 think aloud interviews across a range of timepoints.[41] This think aloud technique will be undertaken either via telephone or face to face. The feasibility of collecting resource use data through nursing home records will be assessed, and the cost of the interventions will be estimated, for use in a full evaluation.

Process Evaluation

The process evaluation elements of the study (Table 5) will address staff members' perceptions of Namaste Care (intervention arm) or perceptions of the effectiveness of usual care (control arm) using interviews approximately 24 weeks after the first resident is recruited at the nursing home. Family carers' perceptions of Namaste Care (intervention arm) or carers' perceptions of the effectiveness of usual care (control arm) will be ascertained using interviews between 16-24 weeks after the first resident is recruited at the nursing home.

To assess the fidelity, acceptability and appropriateness of Namaste Care (intervention arm) or assess effectiveness of usual care (Control arm) observation will be conducted at approximately 2

For peer review only - http://bmjopen.bmj.com/site/about/guidelines.xhtml

weeks, 4 weeks and 24 weeks after the start of the intervention for nursing homes in the intervention arm and approximately 2 weeks and 4 weeks in the control arm.

A data log will be completed by the staff delivering the Namaste Care session throughout the intervention delivery.

Data Management

Data management is provided by the CTRC at the University of Liverpool. Paper based case report forms will be written to record data in a consistent way and ensure, anonymization of the data. Data stored at the CTRC will be checked for missing or unusual values (range checks) and checked for consistency within participants over time. Any suspect data will be returned to the site in the form of data queries. Data query forms will be produced at the CTRC from the trial database and sent either electronically or through the post to a named individual (as listed on the site delegation log). Sites will respond to the queries providing an explanation/resolution to the discrepancies and return the data query forms to CTRC. The forms will then be filed along with the appropriate data collection forms and the appropriate corrections made on the database. The process of database lock, unlock and closure will be followed according to the CTRC policy.

Data Analysis Plan

Three types of data will be analysed: quantitative date from surveys and the actigraphs, qualitative data from interviews and economic data.

Quantitative Analysis

Outcomes at baseline and follow-up will be summarised using descriptive statistics and will be used to make a decision on undertaking a full trial. Analysis of the outcome data will focus on recruitment, response and completion rates, and missing data. Reasons for non-consent and missing outcome data will be reported. Estimates of standard deviation and proxy agreement will be determined, and construct validity estimated intracluster correlation coefficent will be made.

The sleep/activity data from the actigraph will be analysed using summary statistics for the sleep analysis data (sleep/wake ratios, total sleep time, sleep efficiency, wake after sleep onset and total activity); participant's rhythm fragmentation and synchronization will be estimated via Intradaily

For peer review only

Variability (IV) and Interdaily Stability (IS).[42, 43] The actigraph will be used to ascertain the feasibility of use this outcome measure to collect data in a full trial.

Qualitative Analysis

Semi-structured interviews will be audio-recorded, transcribed and anonymised. Framework analysis will be used in the analysis of qualitative data, with data collection, management and analysis rigorously conducted to enable reporting against COREQ guidelines. Group/ individual interviews and observation sessions will be digitally audio-recorded and fully transcribed. NVivo™ will be used to facilitate data management and analysis as this supports framework analysis techniques.

Analysis of Economic Data

Economic assessments of relevant outcome measures will combine qualitative assessments of feasibility of use for the outcome measures gained through the think aloud techniques and more quantitative assessments of agreement between proxies, and assessments of construct validity for the measures.[44] Response and completion rates will be assessed. Constant comparative analytical methods will be used to provide a more in-depth assessment of both the questionnaire completion and respondents' perceptions of the measures in the think aloud interviews.

Unit cost information will be generated using bottom-up costing for the Namaste intervention itself, ensuring that a cost for the intervention will be available in a full trial. Other sources of unit cost information will be identified and collated for use in a future full trial and will be applied to the collected resource use data to enable the preliminary assessment of costs and benefits, and the main cost drivers for a full evaluation... All data will be costed using unit cost data in pounds sterling, and from a single year, as close as possible to the end of the feasibility study.

Public and Patient Involvement

Two carer representatives from the Alzheimer's Society Research Network UK were co-applicants as part of the core study/trial management group. They will be present at all project teleconferences and meetings. A Public Involvement Panel will be established in the north west of England. This will comprise of six to eight members, co-chaired by the PPI co-applicants. The members have personal experience of family members living with dementia in care homes. The panel members will work alongside the research team to assist in different areas of research including reviewing participant

For peer review only - http://bmjopen.bmj.com/site/about/guidelines.xhtml

information sheets and other documentation, five face to face meetings are proposed during the study, and communication between meetings will be by regular updates. There will also be PPI representation on the research advisory group and Trial Steering Committee.

Monitoring and Trial Management

For this research population there is a relatively high risk of death, hospitalisation or progression of disease for participants during the course of the study but which are not anticipated to be related to the receipt of the intervention. This level and type of risk will be treated as an acceptable risk for the purposes of the study and will not constitute adverse events (AE) or serious adverse events (SAE) unless concern is raised by anyone associated with the study that these events could be directly related to participation in this study.

The Trial Management Group, is responsible for 1) protocol completion, 2) obtaining ethical approval for Phases 1 and 2, 3) obtaining ethical approval for Phase 3 plus nursing home approval process; 4) appointing and facilitating the Trial Steering Committee; 5) working with the dissemination partners. The group will meet for a 'kick off' meeting face to face at the start of the project. Thereafter there will be monthly teleconferences and twice yearly face to face meetings. The Trial Steering Committee (TSC), with an independent chair, will provide overall supervision of the trial including trial progress and participant safety. Membership will be drawn from experts in health services research, nursing home research, and PPI. They will meet prior to the start of the trial phase and then twice during the second year of the project. The TSC will have the role of a traditional Data Monitoring Committee as this a feasibility study with a low risk intervention. A TSC charter based on the guidelines published by the NIHR will be used to identify the remit of the TSC committee. An International Advisory Group will also be established to provide external expert advice on the overall progress of the study.

DISCUSSION

This protocol describes the Namaste Care programme for residents with advanced dementia who are living in nursing care homes. The Namaste Care programme is a multi-sensory care programme conducted on a daily basis in a group setting. This study will provide information on implementation, cost and acceptability of a defined intervention. In addition, this study will provide information on usefulness, practicality and acceptability of the selected outcome measures and processes used in

For peer review only - http://bmjopen.bmj.com/site/about/guidelines.xhtml

this study. In conclusion, the findings of this study will informal future research on the Namaste Care programme in nursing care homes.

ETHICS AND DISSEMINATION

The study has been approved by the Wales Research Ethics Committee 5 (Ref: 17/WA/0378). As resident's eligible for the study will lack capacity to consent, consent for residents will be taken from either a personal consultee or a nominated consultee following the Mental Capacity Act (2005) guidance.[28, 45] A procedure for reporting issues of concern in the care setting has been written.

The following dissemination channels will be used: a project website (www.namastetrial.org.uk), a leaflet summarising the study, summaries of findings, publications/articles for general as well as scientific media and social media such as Twitter (@namasteresearch). All publications will follow the relevant reporting guidelines for reviews and trials.[46]

For peer review only

For peer review only - http://bmjopen.bmj.com/site/about/guidelines.xhtml

Table 1. Summary of resident data collected by care home staff, outcome measures and time schedule

Data collected and tool used		Pre-intervention	Monthly	At 6 months or death	
Socio-demographics	Age, gender, ethnicity, existing medical conditions, Stage of dementia on FAST score	x	x	x	
Quality of dying	Measure to assess quality of death using CAD-EOLD[47, 48]	x	x	x	
Quality of Life of the person with dementia	EQ-5D-5L[40] self-rated health index and visual analogue scale of current health state	x	x	x	
Neuropsychiatric Inventory	Measure to assess psychitriatic state of resident using NPI-Q[38]	x	x	x	
Pain	Measure to assess level of pain using PAIN-AD[35]	x	x	x	
Quality of life	EQ-5D-5L	x	x	x	
ICECAP Supportive Care Measure	Health economic measure using ICEPCAP-SCM[38]	x	x	x	
ICECAP-O measure	Health economic measure using ICEPCAP-O[49]	x	x	x	-
Cohen-Mansfield Agitation Inventory	Measure to assess resident agitation[36]	x	x	x	
ICECAP Supportive Care Measure using Think Aloud	Health economic measure using ICEPCAP-SCM using Think Aloud	x	x	x	
ICECAP-O measure using Think Aloud	Health economic measure using ICECAP-O using Think Aloud	x	x	x	

For peer review only

Table 2. Summary of informal carer data collected, as assessed by informal carers, outcome measures and time schedule				
**Data collected and tool used**		**Baseline**	**At 1 Month**	**At 6 months or death**
Socio-demographics	Age, gender, ethnicity, existing medical conditions	x	-	-
Service use in the prior month	Client Service Receipt Inventory (CSRI)[50]. Calculates service and total care costs	x	x	x
Quality of life of the carer	EQ-5D-5L	x	x	x
Satisfaction with Care	SWC-EOLD[34]	x	x	x
Close person measure of health economic evaluation	Health economic evaluation using ICECAP-CPM	x	x	x
Close person measure of health economic evaluation	Health economic evaluation using ICEPCAP-CPM completing using Think Aloud	x	x	x

For peer review only - http://bmjopen.bmj.com/site/about/guidelines.xhtml

Table 3. Summary of staff data collected as assessed by care home staff: outcome measures and time schedule

Data collected and tool used		Pre-intervention	Monthly only	At 6 months	Post intervention
Staff socio-demographics	Age, gender, ethnicity	X	-	-	-
Staff work characteristics	Highest qualification, role in care home, length of service	X	-	-	-
Organizational support for person-centered care	The Person-Centred Care Assessment Tool (P-CAT)[32]	X	-	-	-
Organisational support for readiness for change	The Alberta Context Tool Questionnaire[51]	X	-	-	-

For peer review only

For peer review only - http://bmjopen.bmj.com/site/about/guidelines.xhtml

Table 4. Summary of nursing care home level data collected, outcome measures, time schedule and the type of person assessing the outcome measure

Data collected and tool used		Pre-intervention	Monthly only	At 6 months	Post intervention
Care home occupancy level	Number of available beds to new residents	S	-	-	-
Cost of living in the care home	Fees to live in the care home	S	-	-	-
Contributions from local government	Fees paid by the local government for each resident	S	-	-	-
Staffing levels	Number and type of staff	S	-	-	-
Number of GP practices the care home works with	Number of GP practices the care home works with	S	-	-	-
Number of GPs the care home works with	Number of GPs the care home works with	S	-	-	-
Level of need of residents in the care home	Amount of support each resident needs	S	-	-	-
Staff turnover and sickness levels	Number of staff in the care home and monthly sickness record	S	S	-	-
Ambulances and hospital use	Number and length of hospital admissions (days) , A&E attendances and readmissions	S	S	S	-
Number of hospital admissions	Respiratory infections, urinary tract infections, dehydration, congestive heart failure?	S	S	S	-
Out of hours GP contacts	GP visits or telephone contact	R	R	R	R

Measure assessed by S: care home staff; R: researcher

For peer review only

For peer review only - http://bmjopen.bmj.com/site/about/guidelines.xhtml

Table 5. Data collected as part of the process evaluation.

Outcome Measures or rationale for data collection	Data collected through	Time of data collection
To assess carers' perceptions of Namaste Care (intervention arm) or carers' perceptions of the effectiveness of usual care (control arm)	Interviews conducted by the researcher	Approximately 16 – 24 weeks after the first resident is recruited at the nursing home.<br>(If a resident dies during the trial then the informal carer will be approached at least 8 weeks after the resident's death)
Staff members' perceptions of Namaste Care (intervention arm) or perceptions of the effectiveness of usual care (control arm)	Interviews conducted by the researcher	Approximately 24 weeks after the first resident is recruited at the nursing home
To assess the fidelity, acceptability and appropriateness of Namaste Care (intervention arm) or assess effectiveness of usual care (Control arm)	Observations conducted by the researcher	Approximately 2 weeks, 4 weeks and 24 weeks after the start of the intervention for nursing homes in the intervention arm<br><br>Approximately 2 weeks and 4 weeks after the first resident is recruited for nursing homes in the control arm
To assess the fidelity, acceptability and appropriateness of the Namaste Care (intervention arm)	Data log completed by the staff delivering the Namaste Care session	Throughout the intervention

For peer review only - http://bmjopen.bmj.com/site/about/guidelines.xhtml

REFERENCES

1. Prince M, Bryce R, Albanese E, et al. The global prevalence of dementia: A systematic review and metaanalysis. *Alzheimers & Dementia* 2013;9(1):63-75. doi: 10.1016/j.jalz.2012.11.007
2. Reisberg B. FUNCTIONAL ASSESSMENT STAGING (FAST). *Psychopharmacology Bulletin* 1988;24(4):653-59.
3. Mitchell SL, Teno JM, Kiely DK, et al. The Clinical Course of Advanced Dementia. *New England Journal of Medicine* 2009;361(16):1529-38. doi: 10.1056/NEJMoa0902234
4. Small N. Living well until you die - Quality of care and quality of life in palliative and dementia care. In: Weller NJ, Rattan SIS, eds. Healthy Aging and Longevity2007:194-203.
5. van der Steen JT, Radbruch L, Hertogh C, et al. White paper defining optimal palliative care in older people with dementia: A Delphi study and recommendations from the European Association for Palliative Care. *Palliative Medicine* 2014;28(3):197-209. doi: 10.1177/0269216313493685
6. van der Steen JT, Goodman C. What research we no longer need in neurodegenerative disease at the end of life: The case of research in dementia. *Palliative Medicine* 2015;29(3):189-92. doi: 10.1177/0269216315569998
7. Vink AC, Birks JS, Bruinsma MS, et al. Music therapy for people with dementia. *Cochrane Database Syst Rev* 2004(3):Cd003477. doi: 10.1002/14651858.CD003477.pub2 [published Online First: 2004/07/22]
8. Vasionyte I, Madison G. Musical intervention for patients with dementia: a meta-analysis. *Journal of Clinical Nursing* 2013;22(9-10):1203-16. doi: 10.1111/jocn.12166
9. McDermott O, Crellin N, Ridder HM, et al. Music therapy in dementia: a narrative synthesis systematic review. *International Journal of Geriatric Psychiatry* 2013;28(8):781-94. doi: 10.1002/gps.3895
10. Ueda T, Suzukamo Y, Sato M, et al. Effects of music therapy on behavioral and psychological symptoms of dementia: A systematic review and meta-analysis. *Ageing Research Reviews* 2013;12(2):628-41. doi: 10.1016/j.arr.2013.02.003
11. Viggo Hansen N, Jorgensen T, Ortenblad L. Massage and touch for dementia. *Cochrane Database Syst Rev* 2006(4):Cd004989. doi: 10.1002/14651858.CD004989.pub2 [published Online First: 2006/10/21]
12. Moyle W, Murfield JE, O'Dwyer S, et al. The effect of massage on agitated behaviours in older people with dementia: a literature review. *Journal of Clinical Nursing* 2013;22(5-6):601-10. doi: 10.1111/j.1365-2702.2012.04234.x
13. Li JX, Porock D. Resident outcomes of person-centered care in long-term care: A narrative review of interventional research. *International Journal of Nursing Studies* 2014;51(10):1395-415. doi: 10.1016/j.ijnurstu.2014.04.003
14. Livingston G, Kelly L, Lewis-Holmes E, et al. Non-pharmacological interventions for agitation in dementia: systematic review of randomised controlled trials. *British Journal of Psychiatry* 2014;205(6):436-42. doi: 10.1192/bjp.bp.113.141119
15. Turner S. Behavioural symptoms of dementia in residential settings: A selective review of non-pharmacological interventions. *Aging & Mental Health* 2005;9(2):93-104. doi: 10.1080/13607860512331339090
16. Kverno KS, Black BS, Nolan MT, et al. Research on treating neuropsychiatric symptoms of advanced dementia with non-pharmacological strategies, 1998-2008: a systematic literature review. *International Psychogeriatrics* 2009;21(5):825-43. doi: 10.1017/s1041610209990196
17. Amador S, Goodman C, King D, et al. Exploring resource use and associated costs in end-of-life care for older people with dementia in residential care homes. *International Journal of Geriatric Psychiatry* 2014;29(7):758-66. doi: 10.1002/gps.4061

For peer review only - http://bmjopen.bmj.com/site/about/guidelines.xhtml

18. Simard J, Volicer L. Effects of Namaste Care on residents who do not benefit from usual activities. *Am J Alzheimers Dis Other Demen* 2010;25(1):46-50. doi: 10.1177/1533317509333258 [published Online First: 2009/04/01]
19. Stacpoole M, Hockley J, Thompsell A, et al. The Namaste Care programme can reduce behavioural symptoms in care home residents with advanced dementia. *International Journal of Geriatric Psychiatry* 2015;30(7):702-09. doi: 10.1002/gps.4211
20. Stacpoole M, Hockley J, Thompsell A, et al. Implementing the Namaste Care Program for residents with advanced dementia: exploring the perceptions of families and staff in UK care homes. *Annals of Palliative Medicine* 2017;6(4):327-39. doi: 10.21037/apm.2017.06.26
21. Stacpoole M, Thompsell A. OA25 The namaste care programme can enrich quality of life for people with advanced dementia and those who care for them without additional resources. *BMJ Support Palliat Care* 2015;5 Suppl 1:A8. doi: 10.1136/bmjspcare-2015-000906.25 [published Online First: 2015/05/12]
22. Wen A, Wen A. Behavioral Symptoms Among Patients Before and After Implementation of a Specialized Advanced Dementia Care Program in a Nursing Home. *Journal of the American Medical Directors Association* 2014;15(3):B16. doi: 10.1016/j.jamda.2013.12.043
23. Fullarton J, Volicer L. Reductions of Antipsychotic and Hypnotic Medications in Namaste Care. *Journal of the American Medical Directors Association* 2013;14(9):708-09. doi: 10.1016/j.jamda.2013.06.002
24. Simard J. The end of life Namaste Care program for people with dementia: Health professional press 2013.
25. Stacpoole M, Thompsell A, Hockley J. Toolkit for implementing the Namaste Care programme for people with advanced dementia living in care homes: St Christopher's Hospice; [Available from: https://www.stchristophers.org.uk/wp-content/uploads/2016/03/Namaste-Care-Programme-Toolkit-06.04.2016.pdf accessed 26 July 2018.
26. Hsu MH, Flowerdew R, Parker M, et al. Individual music therapy for managing neuropsychiatric symptoms for people with dementia and their carers: a cluster randomised controlled feasibility study. *Bmc Geriatrics* 2015;15 doi: 10.1186/s12877-015-0082-4
27. Stow R, Ives N, Smith C, et al. A cluster randomised feasibility trial evaluating nutritional interventions in the treatment of malnutrition in care home adult residents. *Trials* 2015;16 doi: 10.1186/s13063-015-0952-2
28. Association BM. Mental Capacity Act Tool kit: BMA; 2008 [Available from: https://www.bma.org.uk/-/media/files/pdfs/practical%20advice%20at%20work/ethics/mental-capacity-act-toolkit-2016.pdf accessed 26 July 2018.
29. Hoffmann TC, Glasziou PP, Boutron I, et al. Better reporting of interventions: template for intervention description and replication (TIDieR) checklist and guide. *BMJ : British Medical Journal* 2014;348 doi: 10.1136/bmj.g1687
30. Volicer L, Hurley AC, Blasi ZV. Scales for evaluation of end-of-life care in dementia. *Alzheimer Disease & Associated Disorders* 2001;15(4):194-200. doi: 10.1097/00002093-200110000-00005
31. Weiner MF, Martin-Cook K, Svetlik DA, et al. The quality of life in late-stage dementia (QUALID) scale. *J Am Med Dir Assoc* 2000;1(3):114-6. [published Online First: 2003/06/24]
32. Edvardsson D, Innes A. Measuring Person-centered Care: A Critical Comparative Review of Published Tools. *Gerontologist* 2010;50(6):834-46. doi: 10.1093/geront/gnq047
33. Kaufer DI, Cummings JL, Ketchel P, et al. Validation of the NPI-Q, a brief clinical form of the neuropsychiatric inventory. *Journal of Neuropsychiatry and Clinical Neurosciences* 2000;12(2):233-39. doi: 10.1176/appi.neuropsych.12.2.233
34. van Soest-Poortvliet MC, van der Steen JT, Zimmerman S, et al. Selecting the Best Instruments to Measure Quality of End-of-Life Care and Quality of Dying in Long Term Care. *Journal of the*

For peer review only - http://bmjopen.bmj.com/site/about/guidelines.xhtml

American Medical Directors Association 2013;14(3):179-86. doi: 10.1016/j.jamda.2012.09.019

35. Warden V, Hurley AC, Volicer L. Development and psychometric evaluation of the Pain Assessment in Advanced Dementia (PAINAD) scale. *J Am Med Dir Assoc* 2003;4(1):9-15. doi: 10.1097/01.jam.0000043422.31640.f7 [published Online First: 2003/06/17]
36. Werner P, Cohen-Mansfield J, Koroknay V, et al. The impact of a restraint-reduction program on nursing home residents. *Geriatr Nurs* 1994;15(3):142-6. [published Online First: 1994/05/01]
37. Wood S, Cummings JL, Hsu MA, et al. The use of the neuropsychiatric inventory in nursing home residents - Characterization and measurement. *American Journal of Geriatric Psychiatry* 2000;8(1):75-83.
38. Coast J, Flynn TN, Natarajan L, et al. Valuing the ICECAP capability index for older people. *Social Science & Medicine* 2008;67(5):874-82. doi: 10.1016/j.socscimed.2008.05.015
39. Grewal I, Lewis J, Flynn T, et al. Developing attributes for a generic quality of life measure for older people: Preferences or capabilities? *Social Science & Medicine* 2006;62(8):1891-901. doi: 10.1016/j.socscimed.2005.08.023
40. Herdman M, Gudex C, Lloyd A, et al. Development and preliminary testing of the new five-level version of EQ-5D (EQ-5D-5L). *Quality of Life Research* 2011;20(10):1727-36. doi: 10.1007/s11136-011-9903-x
41. Schildmann EK, Groeneveld EI, Denzel J, et al. Discovering the hidden benefits of cognitive interviewing in two languages: The first phase of a validation study of the Integrated Palliative care Outcome Scale. *Palliative Medicine* 2016;30(6):599-610. doi: 10.1177/0269216315608348
42. Goncalves BS, Cavalcanti PR, Tavares GR, et al. Nonparametric methods in actigraphy: An update. *Sleep Sci* 2014;7(3):158-64. doi: 10.1016/j.slsci.2014.09.013 [published Online First: 2015/10/21]
43. vanSomeren EJW, Hagebeuk EEO, Lijzenga C, et al. Circadian rest-activity rhythm disturbances in Alzheimer's disease. *Biological Psychiatry* 1996;40(4):259-70. doi: 10.1016/0006-3223(95)00370-3
44. Patten S. Health Measurement Scales: A Practical Guide to Their Development and Use, 4th Edition. *Canadian Journal of Psychiatry-Revue Canadienne De Psychiatrie* 2011;56(3):187-88.
45. Mental Capacity Act. England and Wales, 2005.
46. Moher D, Schulz KF, Altman DG. The CONSORT statement: revised recommendations for improving the quality of reports of parallel-group randomised trials. *Lancet (London, England)* 2001;357(9263):1191-4. [published Online First: 2001/04/27]
47. Kiely DK, Volicer L, Teno J, et al. The validity and reliability of scales for the evaluation of end-of-life care in advanced dementia. *Alzheimer Disease & Associated Disorders* 2006;20(3):176-81. doi: 10.1097/00002093-200607000-00009
48. van der Steen JT, Sampson EL, Van den Block L, et al. Tools to Assess Pain or Lack of Comfort in Dementia: A Content Analysis. *Journal of Pain and Symptom Management* 2015;50(5):659-U250. doi: 10.1016/j.jpainsymman.2015.05.015
49. Makai P, Brouwer WBF, Koopmanschap MA, et al. Capabilities and quality of life in Dutch psycho-geriatric nursing homes: an exploratory study using a proxy version of the ICECAP-O. *Quality of Life Research* 2012;21(5):801-12. doi: 10.1007/s11136-011-9997-1
50. Beecham J, Knapp M. Costing psychiatric interventions, in G. Thornicroft (ed.) Measuring Mental Health Needs. 2nd ed: Gaskell 2001:200-224.
51. Estabrooks CA, Squires JE, Cummings GG, et al. Development and assessment of the Alberta Context Tool. *BMC Health Services Research* 2009;9:234-34. doi: 10.1186/1472-6963-9-234

For peer review only - http://bmjopen.bmj.com/site/about/guidelines.xhtml

FOOTNOTES

Author's contribution: KF, GPA, FB, GB, JC, CG, JK, NP and CW were involved in the conception and design of the trial. SP and KF were involved in the drafting of the article GPA, FB, GB, JC, LD, CG, BH, JK, NP and CW, were involved in critical revision of the article for important intellectual content. All authors were involved in the final approval of the manuscript.

Funding statement: This work was supported by the National Institute of Health Research, HTA programme grant number 15/10/11.The views expressed in this publication are those of the author(s) and not necessarily those of the MRC, NHS, NIHR or the Department of Health and Social Care.

Competing interests statement: None declared.

Ethics approval: Wales Research Ethics Committee 5, UK.

For peer review only

For peer review only - http://bmjopen.bmj.com/site/about/guidelines.xhtml

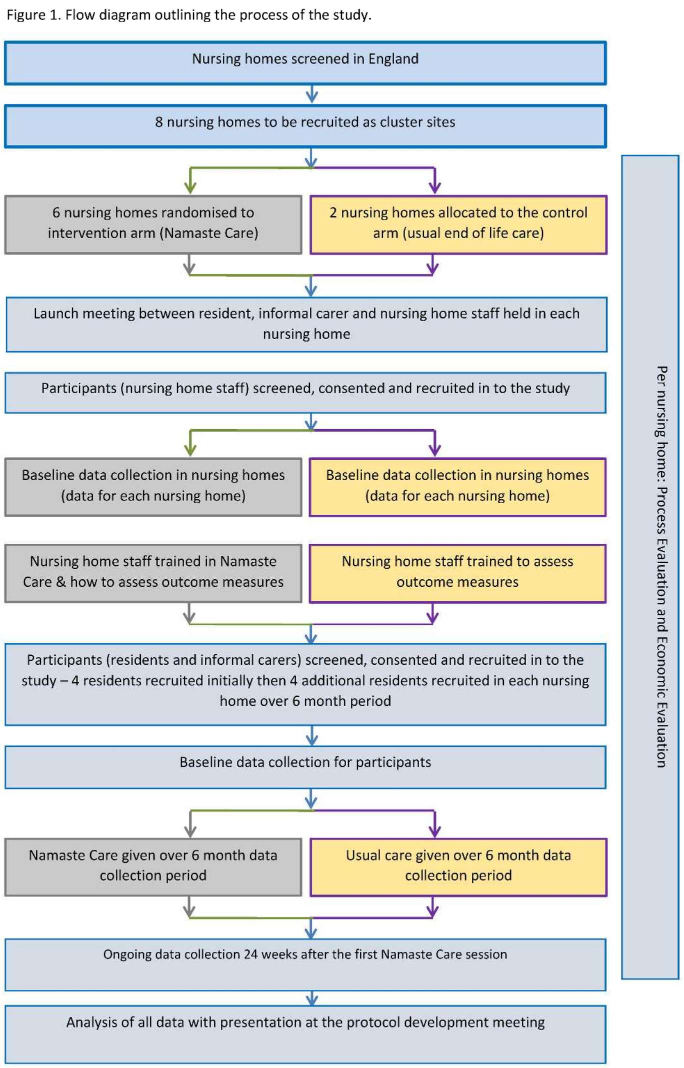

Figure 1. Flow diagram outlining the process of the study

BMJ Open

Namaste Care in nursing care homes with people with advanced dementia: protocol for a feasibility randomised controlled trial

Journal:	*BMJ Open*
Manuscript ID	bmjopen-2018-026531.R1
Article Type:	Protocol
Date Submitted by the Author:	24-Sep-2018
Complete List of Authors:	Froggatt, Katherine ; Lancaster University Faculty of Health and Medicine,<br>Patel, Shakil; Lancaster University Faculty of Health and Medicine<br>Perez Algorta, Guillermo; Lancaster University Faculty of Health and Medicine<br>Bunn, Frances; University of Herfordshire, Department of Health and Human Sciences<br>Burnside, Girvan; University of Liverpool<br>Coast, Joanna; University of Bristol<br>Dunleavy, Lesley; Lancaster University Faculty of Health and Medicine<br>Goodman, Claire; University of Hertfordshire, Centre for Research in Primary and Community Care<br>Hardwick, Ben; University of Liverpool<br>Kinley, Julie; St Chrisptophers Hospice<br>Preston, Nancy J.; Univ Lancaster<br>Walshe, Catherine; Lancaster University
**Primary Subject Heading**:	Palliative care
Secondary Subject Heading:	Geriatric medicine
Keywords:	Dementia < NEUROLOGY, Namaste Care, Feasibility study, Randomised controlled trial, PALLIATIVE CARE, Nursing care homes

SCHOLARONE™
Manuscripts

Namaste Care in nursing care homes with people with advanced dementia: protocol for a feasibility randomised controlled trial

Katherine Froggatt[1], Shakil Patel[1], Guillermo Perez Algorta[1], Frances Bunn[2], Girvan Burnside[4], Joanna Coast[3], Lesley Dunleavy[1], Claire Goodman[2], Ben Hardwick[4], Julie Kinley[5], Nancy Preston[1], Catherine Walshe[1].

1. Lancaster University. 2. University of Hertfordshire. 3. University of Bristol. 4. The University of Liverpool. 5. St. Christopher's Hospice.

Froggatt, Katherine k.froggatt@lancaster.ac.uk; Patel, Shakil s.patel16@lancaster.ac.uk; Perez Algorta, Guillermo g.perezalgorta@lancaster.ac.uk; Frances Bunn f.bunn@herts.ac.uk; Girvan Burnside g.burnside@liverpool.ac.uk; Jo Coast jo.coast@bristol.ac.uk; Dunleavy, Lesley l.dunleavy@lancaster.ac.uk; Claire Goodman c.goodman@herts.ac.uk,Ben Hardwick b.hardwick1@liverpool.ac.uk; Julie Kinley julie@kinley.me.uk; Preston, Nancy n.j.preston@lancaster.ac.uk; Walshe, Catherine c.walshe@lancaster.ac.uk

Corresponding author

Katherine Froggatt

Word Count – 4511 without references

For peer review only - http://bmjopen.bmj.com/site/about/guidelines.xhtml

ABSTRACT

Introduction:

Many people living with advanced dementia live and die in nursing care homes. The quality of life, care and dying experienced by these people is variable. Namaste Care is a multi-sensory programme of care developed for people with advanced dementia. Whilst there is emerging evidence that Namaste Care may be beneficial for people with dementia, there is a need to conduct a feasibility study to establish the optimum way of delivering this complex intervention and whether benefits can be demonstrated in end of life care, for individuals and service delivery. The aim of the study is to ascertain the feasibility of conducting a full trial of the Namaste Care intervention.

Methods and analysis:

A feasibility study, comprising a parallel, two-arm, multi-centre cluster controlled randomised trial with embedded process and economic evaluation. Nursing care homes (total of 8) who deliver care to those with advanced dementia will be randomly allocated to intervention (delivered at nursing care home level) or control. Three participant groups will be recruited: residents with advanced dementia; informal carers of a participating resident and nursing care home staff. Data will be collected for 6 months. Feasibility objectives concern the recruitment and sampling of nursing homes, residents, informal carers and staff; the selection and timing of primary (quality of dying and quality of life) and secondary clinical outcome measures (person centeredness, symptom presence, agitation, quality of life, resource use and costs and residents' activity monitored using actigraphy). Acceptability, fidelity and sustainability of the intervention will be assessed using semi-structured interviews with staff and informal carers

Ethics and dissemination: This protocol has been approved by NHS Wales Research Ethics Committee 5 (Ref: 17/WA0378). Dissemination plans include working with a public involvement panel, through a website (wwww.namastetrial.org.uk), social media, academic and practice conferences and via peer reviewed publications.

Registration: ISRCTN14948133

For peer review only - http://bmjopen.bmj.com/site/about/guidelines.xhtml

Strengths and Limitations of this study

- Intervention trialled is based on a theoretical model of how the intervention works, drawn from current evidence base, and consultation with care home staff, family and experts
- PPI will greatly inform the ongoing development of the research design and delivery and assist in recruitment, analysis and dissemination
- Both proxy and objective measures will be measured with this hard to research population
- Blinding is not possible, due to the nature of the intervention
- The study will not provide data on the effectiveness of the intervention, but will indicate if a further trial to establish effectiveness is feasible

For peer review only

INTRODUCTION

Background

Dementia is a life limiting condition, with a median survival, decreasing with age, of 6.7 to 1.9 years.[1] In advanced dementia, an individual requires full assistance with care, is chair or bedbound, doubly incontinent and no longer able to communicate verbally (FAST scale 6-7).[2] People with dementia often experience a poor quality of death, preceded by a period of poor quality of life, with over and under treatment occurring.[3-5] There is an increasing urgency for appropriate care that will ensure a good quality of life and dying are achieved.[5, 6]

Evidence for therapeutic healthcare interventions for people with advanced dementia is limited. Reviews of therapies such as music therapy indicate mixed outcomes for people with dementia, with a Cochrane review identifying equivocal evidence.[7] More recent reviews of therapeutic interventions have identified large positive effects on behavioural, cognitive and physiological outcomes,[8] to moderate effects on anxiety with small effects on behavioural symptoms and evidence for short term improvement in mood and reduction in behavioural disturbance.[9, 10] In a Cochrane review of touch therapies, some evidence of an effect was identified, but not specifically for people with advanced dementia.[11] A recent review indicated that massage reduced levels of agitation.[12] Interventions supporting person-centred care have been shown to reduce agitation and behavioural disturbance. There is some evidence for individualised interventions, within a bio-psychosocial framework, improving behavioural symptoms.[13-15]

Interventions with a single focus on reducing pain, physical symptoms or specific behavioural disturbances have been found to be effective.[3] It is recognised that for people with advanced dementia there is a need for interventions that complement and enhance pharmacological interventions. This study addresses the lack of evidence available through completed research, to consider the stage specific efficacy of non-pharmacological interventions.[16] There is also a need for practical interventions that staff can learn to deliver which allow them to provide person-centred care.

Palliative and end of life care interventions for people with dementia that emphasise a person-centred philosophy, and use co-design approaches, are being developed and tested.[17] Namaste Care is one such intervention. Non-randomised research studies have identified that Namaste Care at the end of life reduces the severity of behavioural and physical symptoms and occupational disruptiveness and may have an impact on social interaction, delirium and agitation.[18-22] The potential for cost savings with respect to reduced psychotropic medication use has also been indicated.[19, 23] Qualitative evidence suggests greater

For peer review only - http://bmjopen.bmj.com/site/about/guidelines.xhtml

family and staff satisfaction with care.[18] However, none of these studies have compared this intervention with other approaches to palliative and end of life care for this population. We do not yet know the optimum way of delivering this complex intervention and which benefits (including cost-effectiveness) can be demonstrated in end of life care, for individuals and service delivery.

In Phase 1 of this study, a realist review of 85 papers that considered Namaste Care and sensory interventions (such as music therapy or massage) for people with advanced dementia identified three context-mechanism-outcome configurations. This indicated what needs to be in place for Namaste Care to work for this population. The overarching theme was the importance of providing activities that enabled the development of moments of connection for people with advanced dementia. This can occur when the following three elements are in place: provision of structured access to stimulation (social and physical), equipping care home staff to be able to cope with complex variable behaviours, and providing a framework for person-centred care.

Intervention Development

The Namaste Care intervention is already promoted using existing resources. [24, 25] In this study, a four-stage approach to the development and refinement of the intervention resources was used. This entailed: 1. Collating the existing intervention materials and the findings of the realist review to draft an intervention description; 2. Exploring the readability, comprehensibility and utility of the materials with staff unfamiliar with Namaste Care; 3. Using a modified nominal group techniques with people with Namaste Care experience to refine and prioritise the intervention implementation materials; and 4. Final refinement with the study's patient and public involvement panel. This led to production of a 16 page A4 booklet. The booklet included the use of flow charts, graphics and colour coded information supported by infographics, and a training package.

Therefore, we propose undertaking a feasibility cluster controlled randomized trial in a nursing care home context between 01 Jan 2018 to 31 March 2019.

Aims and Objectives

The primary objective of this feasibility study is to ascertain the feasibility of conducting a full trial of the Namaste Care intervention.

For peer review only - http://bmjopen.bmj.com/site/about/guidelines.xhtml

The feasibility issues associated with the research design and data collection processes to enable the design of a full trial to determine the efficacy of Namaste Care are:

a) To understand how best to sample and recruit nursing homes into a cluster randomised controlled trial of Namaste Care;
b) To determine the most appropriate selection, timing and administration of primary and secondary outcome measures for a full cluster randomised controlled trial of Namaste Care against criteria of bias minimization, burden, and acceptability;
c) To establish recruitment, retention and attrition rates at the level of the nursing home and individual resident, informal carer and nursing home staff;
d) To establish the willingness of a large number of nursing homes representing the range of nursing homes, with respect to provider type, size, resident care needs, to participate in a full trial;
e) To assess the acceptability, fidelity and sustainability of the Namaste Care intervention.

Secondary objectives include resident levels of sleep/activity, neuropsychiatric symptoms and pain, informal carer satisfaction with care at the end of life staff care giving experiences and satisfaction with care in end of life care. Health economic and healthcare resource use will also be assessed.

METHODS AND ANALYSIS

Trial Design

A feasibility study consisting of a parallel, two-arm, multi-centre cluster controlled randomised trial design with an embedded process evaluation is to be conducted. The clustering will take place at the nursing care home level. The Namaste Care programme in the intervention arm will be compared with the standard programme of care used in the control homes.

Study Population

Nursing Care Homes

Eight nursing care homes based in the North West of England already using a recognised palliative care programme (for example, Gold Standards Framework for Care Homes, Six Steps to Success or equivalent) will be recruited into the study. Two nursing care homes will be allocated to the control arm whilst six

For peer review only - http://bmjopen.bmj.com/site/about/guidelines.xhtml

For peer review only

nursing care homes will be allocated to the intervention arm. To meet the eligibility criteria, the nursing care home needs to have:

1. at least 30 beds
2. 6 residents who meet the resident eligibility criteria
3. the space to run the Namaste Care programme
4. a manager or a nominated person to act as the Principal Investigator.

A nursing care home will not be eligible to join the study if they:

1. are rated as Needs Improvement or Inadequate in the latest CQC inspection
2. are subject to CQC enforcement notices
3. have already introduced Namaste Care in their nursing care home
4. are currently involved in another research study that conflicts with this study.

Individual Participants

Residents – To meet the resident eligibility criteria, a resident has to:

1. be a permanent resident living in the participating nursing care home
2. lack mental capacity
3. have a formal assessment of advanced dementia based on the Functional Assessment of Staging of Alzheimer's Disease (FAST) score of 6-7 made by the nursing care home manager or another experienced member of staff
4. have a key worker member of staff willing to complete outcome tools.

A resident will be ineligible to participate in the study if the resident:

1. is permanently bedbound
2. is currently or has recently been involved in another research study that conflicts with Namaste Care or with data collection during the course of the Namaste Care study.

Informal carer – To meet the informal carer eligibility criteria, a person who:

1. is 18 years and over

For peer review only - http://bmjopen.bmj.com/site/about/guidelines.xhtml

2. can communicate in English
3. self-defines as a relative or a friend and acts a carer for a resident enrolled to take part in the study.

A person will not be eligible to participate in the study if:

1. their relative or friend is a resident and has not been enrolled in to the study.

Nursing care home staff – To meet the nursing care home eligibility criteria, a person has to be

1. a member of health and social care staff paid to provide care to residents with advanced dementia within participating nursing care homes.

Nursing care home staff will not be eligible to participate in the study if

1. they are in the intervention arm and they have delivered the Namaste Care programme or cared for residents receiving Namaste Care in a nursing care home not involved in this study.

Sample Size and Selection

As the aim of this study is to establish feasibility of a full trial, a formal sample size calculation was not carried out. A sample size of 8 nursing homes (6 intervention and 2 controls) has been selected as it offers a reasonable test of the intervention to assess the feasibility objectives. There have been a range in the sample sizes used in feasibility studies in nursing homes ranging from 2,[26] 6 to 14.[27]

Eligible nursing care homes will be identified through online resources such as the ENRICH database. Following the initial identification, contact will be made with managers of the nursing care home to discuss the study and confirm the eligibility of the nursing care home. Consent for the homes will be assumed when the manager of the facility signs a contract drawn up by the Sponsor, Lancaster University.

Randomisation

The randomisation of participating nursing care homes to either the intervention arm or the control arm will be undertaken by statisticians from the Clinical Trials Research Centre (CTRC) at the University of

For peer review only - http://bmjopen.bmj.com/site/about/guidelines.xhtml

Liverpool randomisation team who will not be involved in the study. Due to the clustered randomisation approach of this study, all study participants will be assigned to the same study arm as the nursing care home they are associated with. The nature of the intervention and its delivery means that it will not be possible to blind nursing homes or staff to the allocation status. If possible, to minimise potential for bias, staff involved in the delivery of the Namaste Care intervention will not be involved in the completion of outcome measures. It will not be possible to blind researchers to the allocation of nursing homes, as the intervention requires changes to the nursing home environment which may be visible to any researcher visiting the facility.

The study flow chart of activities (Figure 1) shows the recruitment process to be followed.

Consent Procedures

Individual Participants

Residents – Potentially eligible residents will be screened by the Principal Investigator and the senior care team at each nursing care home. Consent for the eligible residents will be sought from a personal consultee of the resident in the first instance. If a person consultee does not reply within month of been given the invitation pack then assent will be taken from either a nominated consultee or the process used by the nursing care home in question. Once permission is granted by the personal consultee, members of the research team will discuss the study with the personal consultee and gain assent for residents to take part in the study. Process consent will also be considered for the resident participant group.[28] Therefore, if a resident shows signs of not wanting to take part in the Namaste Care session they will be allowed to miss the Namaste Care session and still continue in the trial.

Informal carer – The informal carers of residents enrolled to the study will be identified by the Principal Investigator and the senior care team at each nursing care home and invited to consent to complete questionnaires and participate in a qualitative interview.

Nursing care home staff – Nursing care home staff interested in taking part in the Namaste Care study will be identified by the nursing care home manager. Upon identification, researchers will discuss the study with the identified staff members and obtain written consent from each staff member.

A research lead will be appointed in each nursing care home. The research lead will be tasked with ensuring the paperwork associated with clinical research and the Investigator Site File is maintained. The

For peer review only - http://bmjopen.bmj.com/site/about/guidelines.xhtml

research lead, and the Principal Investigator from the intervention sites and the control sites will be invited to a training day for guidance on selection of participants and completion of data collection forms and maintaining the Investigator Site File.

Participants will be followed for six months after the commencement of the Namaste Care intervention in each nursing care home in the intervention arm or after the recruitment of the first four residents in the nursing care home for sites in the control arm.

Intervention

The intervention is a programme of care (Namaste Care), delivered in the intervention care homes by care staff working in the facility. The following description uses the TIDieR guidelines for intervention description (items 1-9).[29]

Namaste Care seeks to give comfort and pleasure to people with advanced dementia through engagement, meaningful and creative activities as well as sensory stimulation to reflect the resident's 'life story'[24]. Supporting resource materials have been developed which provide the following guidance regarding the implementation of Namaste Care programme.

- The Namaste Care sessions should be undertaken within a designated space in the nursing home. This space could be within another room, or a room which is used for other purposes
- The environment of the designated space must be made 'special' and should enable a feeling of calm i.e. welcoming and homely, with natural or slightly dimmed lighting, perhaps attractive scents, such as lavender from an aromatherapy diffuser, and with soft music playing
- The Namaste Care sessions should be undertaken in a group setting
- Food and drink should be offered to the residents
- A minimum of two nursing home staff members or volunteers should be present to run the Namaste Care sessions
- The duration and frequency of Namaste Care delivery as proposed by its originator (two hours a day, twice a day, seven days a week) will be promoted.[24]

Namaste Care champions will be appointed in each nursing care home in the intervention arm. At least two care staff (registered nurses, care assistants or activity coordinators) will attend a one day workshop

For peer review only - http://bmjopen.bmj.com/site/about/guidelines.xhtml

about Namaste Care, led by an experienced external facilitator. A follow up training session will be held at each nursing care home to train more staff and provide advice on preparing the Namaste space.

Prior to the commencement of enrolment, Namaste Care champions (Eligible nursing care homes will be identified in the intervention arm) will be appointed in each nursing care home. The Namaste Care champion will be invited to a training day for guidance on Namaste Care intervention, held at a site away from nursing care homes and undertaken by members of the research team and an external trainer. A follow up training session will be held at each nursing care home.

Control Arm

The care home manager of nursing care homes allocated to the control arm will be asked to continue delivering the usual care programme used in their facility.

Training on the Namaste Care programme will be available to the nursing care homes in the control arm after the study has been completed.

Outcome and Study Measures

We consider two contender primary outcomes for a full trial: (1) quality of dying (dementia) (CAD-EOLD) and (2) quality of life (QUALID) (Table 1 -4). [30, 31]

The secondary outcome measures in this trial (Table 1) will measure: measure person-centeredness, symptom presence, agitation, quality of life, resource use and costs; and sleep and activity using actigraphy.[32-37] Semi-structured interviews with staff and informal carers will assess perceptions of Namaste Care or usual care, assessment of the fidelity, acceptability and appropriateness of Namaste Care or of usual care.

The outcome measures to be used are listed in Tables 1 – 4 and presented based on respondent type i.e. measures for residents (Table 1), informal carers (Table 2), staff (Table 3) and at the level of the nursing care home (Table 4). At the start of the study, descriptive data will be collected for all participating nursing care homes such as ownership and funding model, size, staffing, case mix, staff turnover, staff sickness/absence and geographical location. An interview with the nursing care home manager will also be conducted to ascertain the organisation's readiness for change.

For peer review only

For peer review only

For peer review only - http://bmjopen.bmj.com/site/about/guidelines.xhtml

Table 1. Summary of resident data collected by care home staff, outcome measures and time schedule

Data collected and tool used		Pre-intervention	Monthly	At 6 months or death
Socio-demographics	Age, gender, ethnicity, existing medical conditions, Stage of dementia on FAST score	x	x	x
Quality of dying	Measure to assess quality of death using CAD-EOLD[38, 39]	x	x	x
Quality of Life of the person with dementia	EQ-5D-5L[40] self-rated health index and visual analogue scale of current health state	x	x	x
Neuropsychiatric Inventory	Measure to assess psychitriatic state of resident using NPI-Q[41]	x	x	x
Pain	Measure to assess level of pain using PAIN-AD[35]	x	x	x
Quality of life	EQ-5D-5L	x	x	x
ICECAP Supportive Care Measure	Health economic measure using ICEPCAP-SCM[41]	x	x	x
ICECAP-O measure	Health economic measure using ICEPCAP-O[42]	x	x	x
Cohen-Mansfield Agitation Inventory	Measure to assess resident agitation[36]	x	x	x
ICECAP Supportive Care Measure using Think Aloud	Health economic measure using ICEPCAP-SCM using Think Aloud	x	x	x
ICECAP-O measure using Think Aloud	Health economic measure using ICECAP-O using Think Aloud	x	x	x

For peer review only

For peer review only - http://bmjopen.bmj.com/site/about/guidelines.xhtml

Table 2. Summary of informal carer data collected, as assessed by informal carers, outcome measures and time schedule

**Data collected and tool used**		**Baseline**	**At 1 Month**	**At 6 months or death**
Socio-demographics	Age, gender, ethnicity, existing medical conditions	x	-	-
Service use in the prior month	Client Service Receipt Inventory (CSRI)[43]. Calculates service and total care costs	x	x	x
Quality of life of the carer	EQ-5D-5L	x	x	x
Satisfaction with Care	SWC-EOLD[34]	x	x	x
Close person measure of health economic evaluation	Health economic evaluation using ICECAP-CPM	x	x	x
Close person measure of health economic evaluation	Health economic evaluation using ICEPCAP-CPM completing using Think Aloud	x	x	x

For peer review only - http://bmjopen.bmj.com/site/about/guidelines.xhtml

Table 3. Summary of staff data collected as assessed by care home staff: outcome measures and time schedule

**Data collected and tool used**		**Pre-intervention**	**Monthly only**	**At 6 months [illegible] intervention**
Staff socio-demographics	Age, gender, ethnicity	X	-	-
Staff work characteristics	Highest qualification, role in care home, length of service	X	-	-
Organizational support for person-centered care	The Person-Centred Care Assessment Tool (P-CAT)[32]	X	-	-
Organisational support for readiness for change	The Alberta Context Tool Questionnaire[44]	X	-	-

For peer review only

For peer review only - http://bmjopen.bmj.com/site/about/guidelines.xhtml

Table 4. Summary of nursing care home level data collected, outcome measures, time schedule and the type of person assessing the outcome measure

**Data collected and tool used**		**Pre-intervention**	**Monthly**	**intervention At 6 months only**
Care home occupancy level	Number of available beds to new residents	S	-	-
Cost of living in the care home	Fees to live in the care home	S	-	-
Contributions from local government	Fees paid by the local government for each resident	S	-	-
Staffing levels	Number and type of staff	S	-	-
Number of GP practices the care home works with	Number of GP practices the care home works with	S	-	-
Number of GPs the care home works with	Number of GPs the care home works with	S	-	-
Level of need of residents in the care home	Amount of support each resident needs	S	-	-
Staff turnover and sickness levels	Number of staff in the care home and monthly sickness record	S	S	-
Ambulances and hospital use	Number and length of hospital admissions (days) , A&E attendances and readmissions	S	S	S
Number of hospital admissions	Respiratory infections, urinary tract infections, dehydration, congestive heart failure?	S	S	S
Out of hours GP contacts	GP visits or telephone contact	R	R	R

Measure assessed by S: care home staff; R: researcher

For peer review only - http://bmjopen.bmj.com/site/about/guidelines.xhtml

Data Collection

In this study, the outcome measures and process evaluation data will be gathered via 5 different methods:

1) Questionnaires – The nursing home staff participant group and the informal carer participant group will be asked to complete written questionnaires at timepoints outlined in Tables 1 – 3. The questionnaires for the resident participant group will be proxy completed by nursing care home staff who are key workers for the participating residents. Note, the timeframe for baseline varies depending on the participant group. Data on nursing home level data about engagement with health and social care services will be collected using standardised data collection forms (Table 4)
2) Objective measures – The participating residents will be asked to wear an actigraph watch-like device for 28 days from the baseline visit. This actigraph will be placed on the wrist or ankle of the resident and will be used to continuously measure sleep and activity
3) Interviews - Semi-structured interviews will be undertaken at the baseline with the nursing home manager and at the end of the data collection period with family carers and care staff
4) Observations of the residents will be undertaken intermittently during the delivery of the care programme and during the delivery of usual care in the control sites
5) Data logs will be completed in the intervention sites using a proforma to record intervention delivery.

Feasibility Work for Economic Evaluation

The use of a number of potential outcome measures will be explored in terms of feasibility and acceptability of proxy completion with the particular population, evaluated through the think aloud technique. The chosen measures are included in the NICE recommended measures for health and social care: EQ-5D-5L (5 items), the ICECAP-O (5 items) and the ICECAP-Supportive Care Measure (ICECAP-SCM) (7 items).[40, 41, 45] A think aloud technique will also be used with the ICECAP-O, ICECAP-SCM and ICECAP-CPM tools for a proportion of participants at 2 weeks, 4 weeks and 24 weeks, to obtain 20-30 think aloud interviews across a range of timepoints.[46] This think aloud technique will be undertaken either via telephone or face to face. The feasibility of collecting resource use data through nursing home records will be assessed, and the cost of the interventions will be estimated, for use in a full evaluation.

For peer review only - http://bmjopen.bmj.com/site/about/guidelines.xhtml

Process Evaluation

The process evaluation elements of the study (Table 5) will address staff members' perceptions of Namaste Care (intervention arm) or perceptions of the effectiveness of usual care (control arm) using interviews approximately 24 weeks after the first resident is recruited at the nursing home. Family carers' perceptions of Namaste Care (intervention arm) or carers' perceptions of the effectiveness of usual care (control arm) will be ascertained using interviews between 16-24 weeks after the first resident is recruited at the nursing home.

To assess the fidelity, acceptability and appropriateness of Namaste Care (intervention arm) or assess effectiveness of usual care (Control arm) observation will be conducted at approximately 2 weeks, 4 weeks and 24 weeks after the start of the intervention for nursing homes in the intervention arm and approximately 2 weeks and 4 weeks in the control arm.

A data log will be completed by the staff delivering the Namaste Care session throughout the intervention delivery.

For peer review only

For peer review only - http://bmjopen.bmj.com/site/about/guidelines.xhtml

Table 5. Data collected as part of the process evaluation.

**Outcome Measures or rationale for data collection**	**Data collected through**	**Time of data collection**
To assess carers' perceptions of Namaste Care (intervention arm) or carers' perceptions of the effectiveness of usual care (control arm)	Interviews conducted by the researcher	Approximately 16 – 24 weeks after the first resident is recruited at the nursing home.<br>(If a resident dies during the trial then the informal carer will be approached at least 8 weeks after the resident's death)
Staff members' perceptions of Namaste Care (intervention arm) or perceptions of the effectiveness of usual care (control arm)	Interviews conducted by the researcher	Approximately 24 weeks after the first resident is recruited at the nursing home
To assess the fidelity, acceptability and appropriateness of Namaste Care (intervention arm) or assess effectiveness of usual care (Control arm)	Observations conducted by the researcher	Approximately 2 weeks, 4 weeks and 24 weeks after the start of the intervention for nursing homes in the intervention arm<br><br>Approximately 2 weeks and 4 weeks after the first resident is recruited for nursing homes in the control arm
To assess the fidelity, acceptability and appropriateness of the Namaste Care (intervention arm)	Data log completed by the staff delivering the Namaste Care session	Throughout the intervention

For peer review only - http://bmjopen.bmj.com/site/about/guidelines.xhtml

For peer review only

For peer review only - http://bmjopen.bmj.com/site/about/guidelines.xhtml

Data Management

Data management is provided by the CTRC at the University of Liverpool. Paper based case report forms will be written to record data in a consistent way and ensure, anonymization of the data. Data stored at the CTRC will be checked for missing or unusual values (range checks) and checked for consistency within participants over time. Any suspect data will be returned to the site in the form of data queries. Data query forms will be produced at the CTRC from the trial database and sent either electronically or through the post to a named individual (as listed on the site delegation log). Sites will respond to the queries providing an explanation/resolution to the discrepancies and return the data query forms to CTRC. The forms will then be filed along with the appropriate data collection forms and the appropriate corrections made on the database. The process of database lock, unlock and closure will be followed according to the CTRC policy.

Data Analysis Plan

Three types of data will be analysed: quantitative date from surveys and the actigraphs, qualitative data from interviews and economic data.

Quantitative Analysis

Outcomes at baseline and follow-up will be summarised using descriptive statistics and will be used to make a decision on undertaking a full trial. Analysis of the outcome data will focus on recruitment, response and completion rates, and missing data. Reasons for non-consent and missing outcome data will be reported. Estimates of standard deviation and proxy agreement will be determined, and construct validity estimated intracluster correlation coefficent will be made.

The sleep/activity data from the actigraph will be analysed using summary statistics for the sleep analysis data (sleep/wake ratios, total sleep time, sleep efficiency, wake after sleep onset and total activity); participant's rhythm fragmentation and synchronization will be estimated via Intradaily Variability (IV) and Interdaily Stability (IS).[47, 48] The actigraph will be used to ascertain the feasibility of use this outcome measure to collect data in a full trial.

For peer review only - http://bmjopen.bmj.com/site/about/guidelines.xhtml

Qualitative Analysis

Semi-structured interviews will be audio-recorded, transcribed and anonymised. Framework analysis will be used in the analysis of qualitative data, with data collection, management and analysis rigorously conducted to enable reporting against COREQ guidelines. Group/ individual interviews and observation sessions will be digitally audio-recorded and fully transcribed. NVivo™ will be used to facilitate data management and analysis as this supports framework analysis techniques.

For peer review only

Analysis of Economic Data

Economic assessments of relevant outcome measures will combine qualitative assessments of feasibility of use for the outcome measures gained through the think aloud techniques and more quantitative assessments of agreement between proxies, and assessments of construct validity for the measures.[49] Response and completion rates will be assessed. Constant comparative analytical methods will be used to provide a more in-depth assessment of both the questionnaire completion and respondents' perceptions of the measures in the think aloud interviews.

Unit cost information will be generated using bottom-up costing for the Namaste intervention itself, ensuring that a cost for the intervention will be available in a full trial. Other sources of unit cost information will be identified and collated for use in a future full trial and will be applied to the collected resource use data to enable the preliminary assessment of costs and benefits, and the main cost drivers for a full evaluation. All data will be costed using unit cost data in pounds sterling, and from a single year, as close as possible to the end of the feasibility study.

Public and Patient Involvement

Two carer representatives from the Alzheimer's Society Research Network UK were co-applicants as part of the core study/trial management group. They will be present at all project teleconferences and meetings. A Public Involvement Panel will be established in the north west of England. This will comprise of six to eight members, co-chaired by the PPI co-applicants. The members have personal experience of family members living with dementia in care homes. The panel members will work alongside the research team to assist in different areas of research including reviewing participant information sheets and other

For peer review only - http://bmjopen.bmj.com/site/about/guidelines.xhtml

documentation, five face to face meetings are proposed during the study, and communication between meetings will be by regular updates. There will also be PPI representation on the research advisory group and Trial Steering Committee.

Monitoring and Trial Management

For this research population there is a relatively high risk of death, hospitalisation or progression of disease for participants during the course of the study but which are not anticipated to be related to the receipt of the intervention. This level and type of risk will be treated as an acceptable risk for the purposes of the study and will not constitute adverse events (AE) or serious adverse events (SAE) unless concern is raised by anyone associated with the study that these events could be directly related to participation in this study.

The Trial Management Group, is responsible for 1) protocol completion, 2) obtaining ethical approval for Phases 1 and 2, 3) obtaining ethical approval for Phase 3 plus nursing home approval process; 4) appointing and facilitating the Trial Steering Committee; 5) working with the dissemination partners. The group will meet for a 'kick off' meeting face to face at the start of the project. Thereafter there will be monthly teleconferences and twice yearly face to face meetings. The Trial Steering Committee (TSC), with an independent chair, will provide overall supervision of the trial including trial progress and participant safety. Membership will be drawn from experts in health services research, nursing home research, and PPI. They will meet prior to the start of the trial phase and then twice during the second year of the project. The TSC will have the role of a traditional Data Monitoring Committee as this a feasibility study with a low risk intervention. A TSC charter based on the guidelines published by the NIHR will be used to identify the remit of the TSC committee. An International Advisory Group will also be established to provide external expert advice on the overall progress of the study. There is a data management plan (held by the sponsor) which outlines data storage periods and future access to data.

DISCUSSION

This protocol describes the Namaste Care programme for residents with advanced dementia who are living in nursing care homes. The Namaste Care programme is a multi-sensory care programme conducted on a daily basis in a group setting. This study will provide information on implementation, cost and

For peer review only - http://bmjopen.bmj.com/site/about/guidelines.xhtml

acceptability of a defined intervention. In addition, this study will provide information on usefulness, practicality and acceptability of the selected outcome measures and processes used in this study. In conclusion, the findings of this study will informal future research on the Namaste Care programme in nursing care homes.

For peer review only

ETHICS AND DISSEMINATION

The study has been approved by the Wales Research Ethics Committee 5 (Ref: 17/WA/0378; Ver No 04. Feb 09 2018). As resident's eligible for the study will lack capacity to consent, consent for residents will be taken from either a personal consultee or a nominated consultee following the Mental Capacity Act (2005) guidance.[28, 50] A procedure for reporting issues of concern in the care setting has been written.

The following dissemination channels will be used: a project website (www.namastetrial.org.uk), a leaflet summarising the study, summaries of findings, publications/articles for general as well as scientific media and social media such as Twitter (@namasteresearch). All publications will follow the relevant reporting guidelines for reviews and trials.[51]

For peer review only

For peer review only - http://bmjopen.bmj.com/site/about/guidelines.xhtml

REFERENCES

1. Prince M, Bryce R, Albanese E, et al. The global prevalence of dementia: A systematic review and metaanalysis. *Alzheimers & Dementia* 2013;9(1):63-75. doi: 10.1016/j.jalz.2012.11.007
2. Reisberg B. FUNCTIONAL ASSESSMENT STAGING (FAST). *Psychopharmacology Bulletin* 1988;24(4):653-59.
3. Mitchell SL, Teno JM, Kiely DK, et al. The Clinical Course of Advanced Dementia. *New England Journal of Medicine* 2009;361(16):1529-38. doi: 10.1056/NEJMoa0902234
4. Small N. Living well until you die - Quality of care and quality of life in palliative and dementia care. In: Weller NJ, Rattan SIS, eds. Healthy Aging and Longevity2007:194-203.
5. van der Steen JT, Radbruch L, Hertogh C, et al. White paper defining optimal palliative care in older people with dementia: A Delphi study and recommendations from the European Association for Palliative Care. *Palliative Medicine* 2014;28(3):197-209. doi: 10.1177/0269216313493685
6. van der Steen JT, Goodman C. What research we no longer need in neurodegenerative disease at the end of life: The case of research in dementia. *Palliative Medicine* 2015;29(3):189-92. doi: 10.1177/0269216315569998
7. Vink AC, Birks JS, Bruinsma MS, et al. Music therapy for people with dementia. *Cochrane Database Syst Rev* 2004(3):Cd003477. doi: 10.1002/14651858.CD003477.pub2 [published Online First: 2004/07/22]
8. Vasionyte I, Madison G. Musical intervention for patients with dementia: a meta-analysis. *Journal of Clinical Nursing* 2013;22(9-10):1203-16. doi: 10.1111/jocn.12166
9. McDermott O, Crellin N, Ridder HM, et al. Music therapy in dementia: a narrative synthesis systematic review. *International Journal of Geriatric Psychiatry* 2013;28(8):781-94. doi: 10.1002/gps.3895
10. Ueda T, Suzukamo Y, Sato M, et al. Effects of music therapy on behavioral and psychological symptoms of dementia: A systematic review and meta-analysis. *Ageing Research Reviews* 2013;12(2):628-41. doi: 10.1016/j.arr.2013.02.003
11. Viggo Hansen N, Jorgensen T, Ortenblad L. Massage and touch for dementia. *Cochrane Database Syst Rev* 2006(4):Cd004989. doi: 10.1002/14651858.CD004989.pub2 [published Online First: 2006/10/21]
12. Moyle W, Murfield JE, O'Dwyer S, et al. The effect of massage on agitated behaviours in older people with dementia: a literature review. *Journal of Clinical Nursing* 2013;22(5-6):601-10. doi: 10.1111/j.1365-2702.2012.04234.x
13. Li JX, Porock D. Resident outcomes of person-centered care in long-term care: A narrative review of interventional research. *International Journal of Nursing Studies* 2014;51(10):1395-415. doi: 10.1016/j.ijnurstu.2014.04.003
14. Livingston G, Kelly L, Lewis-Holmes E, et al. Non-pharmacological interventions for agitation in dementia: systematic review of randomised controlled trials. *British Journal of Psychiatry* 2014;205(6):436-42. doi: 10.1192/bjp.bp.113.141119
15. Turner S. Behavioural symptoms of dementia in residential settings: A selective review of non-pharmacological interventions. *Aging & Mental Health* 2005;9(2):93-104. doi: 10.1080/13607860512331339090
16. Kverno KS, Black BS, Nolan MT, et al. Research on treating neuropsychiatric symptoms of advanced dementia with non-pharmacological strategies, 1998-2008: a systematic literature review. *International Psychogeriatrics* 2009;21(5):825-43. doi: 10.1017/s1041610209990196

For peer review only - http://bmjopen.bmj.com/site/about/guidelines.xhtml

17. Amador S, Goodman C, King D, et al. Exploring resource use and associated costs in end-of-life care for older people with dementia in residential care homes. *International Journal of Geriatric Psychiatry* 2014;29(7):758-66. doi: 10.1002/gps.4061
18. Simard J, Volicer L. Effects of Namaste Care on residents who do not benefit from usual activities. *Am J Alzheimers Dis Other Demen* 2010;25(1):46-50. doi: 10.1177/1533317509333258 [published Online First: 2009/04/01]
19. Stacpoole M, Hockley J, Thompsell A, et al. The Namaste Care programme can reduce behavioural symptoms in care home residents with advanced dementia. *International Journal of Geriatric Psychiatry* 2015;30(7):702-09. doi: 10.1002/gps.4211
20. Stacpoole M, Hockley J, Thompsell A, et al. Implementing the Namaste Care Program for residents with advanced dementia: exploring the perceptions of families and staff in UK care homes. *Annals of Palliative Medicine* 2017;6(4):327-39. doi: 10.21037/apm.2017.06.26
21. Stacpoole M, Thompsell A. OA25 The namaste care programme can enrich quality of life for people with advanced dementia and those who care for them without additional resources. *BMJ Support Palliat Care* 2015;5 Suppl 1:A8. doi: 10.1136/bmjspcare-2015-000906.25 [published Online First: 2015/05/12]
22. Wen A, Wen A. Behavioral Symptoms Among Patients Before and After Implementation of a Specialized Advanced Dementia Care Program in a Nursing Home. *Journal of the American Medical Directors Association* 2014;15(3):B16. doi: 10.1016/j.jamda.2013.12.043
23. Fullarton J, Volicer L. Reductions of Antipsychotic and Hypnotic Medications in Namaste Care. *Journal of the American Medical Directors Association* 2013;14(9):708-09. doi: 10.1016/j.jamda.2013.06.002
24. Simard J. The end of life Namaste Care program for people with dementia: Health professional press 2013.
25. Stacpoole M, Thompsell A, Hockley J. Toolkit for implementing the Namaste Care programme for people with advanced dementia living in care homes: St Christopher's Hospice; [Available from: https://www.stchristophers.org.uk/wp-content/uploads/2016/03/Namaste-Care-Programme-Toolkit-06.04.2016.pdf accessed 26 July 2018.
26. Hsu MH, Flowerdew R, Parker M, et al. Individual music therapy for managing neuropsychiatric symptoms for people with dementia and their carers: a cluster randomised controlled feasibility study. *Bmc Geriatrics* 2015;15 doi: 10.1186/s12877-015-0082-4
27. Stow R, Ives N, Smith C, et al. A cluster randomised feasibility trial evaluating nutritional interventions in the treatment of malnutrition in care home adult residents. *Trials* 2015;16 doi: 10.1186/s13063-015-0952-2
28. Association BM. Mental Capacity Act Tool kit: BMA; 2008 [Available from: https://www.bma.org.uk/-/media/files/pdfs/practical%20advice%20at%20work/ethics/mental-capacity-act-toolkit-2016.pdf accessed 26 July 2018.
29. Hoffmann TC, Glasziou PP, Boutron I, et al. Better reporting of interventions: template for intervention description and replication (TIDieR) checklist and guide. *BMJ : British Medical Journal* 2014;348 doi: 10.1136/bmj.g1687
30. Volicer L, Hurley AC, Blasi ZV. Scales for evaluation of end-of-life care in dementia. *Alzheimer Disease & Associated Disorders* 2001;15(4):194-200. doi: 10.1097/00002093-200110000-00005
31. Weiner MF, Martin-Cook K, Svetlik DA, et al. The quality of life in late-stage dementia (QUALID) scale. *J Am Med Dir Assoc* 2000;1(3):114-6. [published Online First: 2003/06/24]
32. Edvardsson D, Innes A. Measuring Person-centered Care: A Critical Comparative Review of Published Tools. *Gerontologist* 2010;50(6):834-46. doi: 10.1093/geront/gnq047

For peer review only - http://bmjopen.bmj.com/site/about/guidelines.xhtml

33. Kaufer DI, Cummings JL, Ketchel P, et al. Validation of the NPI-Q, a brief clinical form of the neuropsychiatric inventory. *Journal of Neuropsychiatry and Clinical Neurosciences* 2000;12(2):233-39. doi: 10.1176/appi.neuropsych.12.2.233
34. van Soest-Poortvliet MC, van der Steen JT, Zimmerman S, et al. Selecting the Best Instruments to Measure Quality of End-of-Life Care and Quality of Dying in Long Term Care. *Journal of the American Medical Directors Association* 2013;14(3):179-86. doi: 10.1016/j.jamda.2012.09.019
35. Warden V, Hurley AC, Volicer L. Development and psychometric evaluation of the Pain Assessment in Advanced Dementia (PAINAD) scale. *J Am Med Dir Assoc* 2003;4(1):9-15. doi: 10.1097/01.jam.0000043422.31640.f7 [published Online First: 2003/06/17]
36. Werner P, Cohen-Mansfield J, Koroknay V, et al. The impact of a restraint-reduction program on nursing home residents. *Geriatr Nurs* 1994;15(3):142-6. [published Online First: 1994/05/01]
37. Wood S, Cummings JL, Hsu MA, et al. The use of the neuropsychiatric inventory in nursing home residents - Characterization and measurement. *American Journal of Geriatric Psychiatry* 2000;8(1):75-83.
38. Kiely DK, Volicer L, Teno J, et al. The validity and reliability of scales for the evaluation of end-of-life care in advanced dementia. *Alzheimer Disease & Associated Disorders* 2006;20(3):176-81. doi: 10.1097/00002093-200607000-00009
39. van der Steen JT, Sampson EL, Van den Block L, et al. Tools to Assess Pain or Lack of Comfort in Dementia: A Content Analysis. *Journal of Pain and Symptom Management* 2015;50(5):659-U250. doi: 10.1016/j.jpainsymman.2015.05.015
40. Herdman M, Gudex C, Lloyd A, et al. Development and preliminary testing of the new five-level version of EQ-5D (EQ-5D-5L). *Quality of Life Research* 2011;20(10):1727-36. doi: 10.1007/s11136-011-9903-x
41. Coast J, Flynn TN, Natarajan L, et al. Valuing the ICECAP capability index for older people. *Social Science & Medicine* 2008;67(5):874-82. doi: 10.1016/j.socscimed.2008.05.015
42. Makai P, Brouwer WBF, Koopmanschap MA, et al. Capabilities and quality of life in Dutch psycho-geriatric nursing homes: an exploratory study using a proxy version of the ICECAP-O. *Quality of Life Research* 2012;21(5):801-12. doi: 10.1007/s11136-011-9997-1
43. Beecham J, Knapp M. Costing psychiatric interventions, in G. Thornicroft (ed.) Measuring Mental Health Needs. 2nd ed: Gaskell 2001:200-224.
44. Estabrooks CA, Squires JE, Cummings GG, et al. Development and assessment of the Alberta Context Tool. *BMC Health Services Research* 2009;9:234-34. doi: 10.1186/1472-6963-9-234
45. Grewal I, Lewis J, Flynn T, et al. Developing attributes for a generic quality of life measure for older people: Preferences or capabilities? *Social Science & Medicine* 2006;62(8):1891-901. doi: 10.1016/j.socscimed.2005.08.023
46. Schildmann EK, Groeneveld EI, Denzel J, et al. Discovering the hidden benefits of cognitive interviewing in two languages: The first phase of a validation study of the Integrated Palliative care Outcome Scale. *Palliative Medicine* 2016;30(6):599-610. doi: 10.1177/0269216315608348
47. Goncalves BS, Cavalcanti PR, Tavares GR, et al. Nonparametric methods in actigraphy: An update. *Sleep Sci* 2014;7(3):158-64. doi: 10.1016/j.slsci.2014.09.013 [published Online First: 2015/10/21]
48. vanSomeren EJW, Hagebeuk EEO, Lijzenga C, et al. Circadian rest-activity rhythm disturbances in Alzheimer's disease. *Biological Psychiatry* 1996;40(4):259-70. doi: 10.1016/0006-3223(95)00370-3
49. Patten S. Health Measurement Scales: A Practical Guide to Their Development and Use, 4th Edition. *Canadian Journal of Psychiatry-Revue Canadienne De Psychiatrie* 2011;56(3):187-88.
50. Mental Capacity Act. England and Wales, 2005.

For peer review only - http://bmjopen.bmj.com/site/about/guidelines.xhtml

51. Moher D, Schulz KF, Altman DG. The CONSORT statement: revised recommendations for improving the quality of reports of parallel-group randomised trials. *Lancet* 2001;357(9263):1191-4. [published Online First: 2001/04/27]

FOOTNOTES

Author's contribution: KF, GPA, FB, GB, JC, CG, JK, NP and CW were involved in the conception and design of the trial. SP and KF were involved in the drafting of the article GPA, FB, GB, JC, LD, CG, BH, JK, NP and CW, were involved in critical revision of the article for important intellectual content. All authors were involved in the final approval of the manuscript.

Funding statement: This work was supported by the National Institute of Health Research, HTA programme grant number 15/10/11.The views expressed in this publication are those of the author(s) and not necessarily those of the MRC, NHS, NIHR or the Department of Health and Social Care.

Competing interests statement: None declared.

Ethics approval: Wales Research Ethics Committee 5, UK.

Figure Legends

Figure 1. Flow diagram outlining the process of the study

For peer review only - http://bmjopen.bmj.com/site/about/guidelines.xhtml

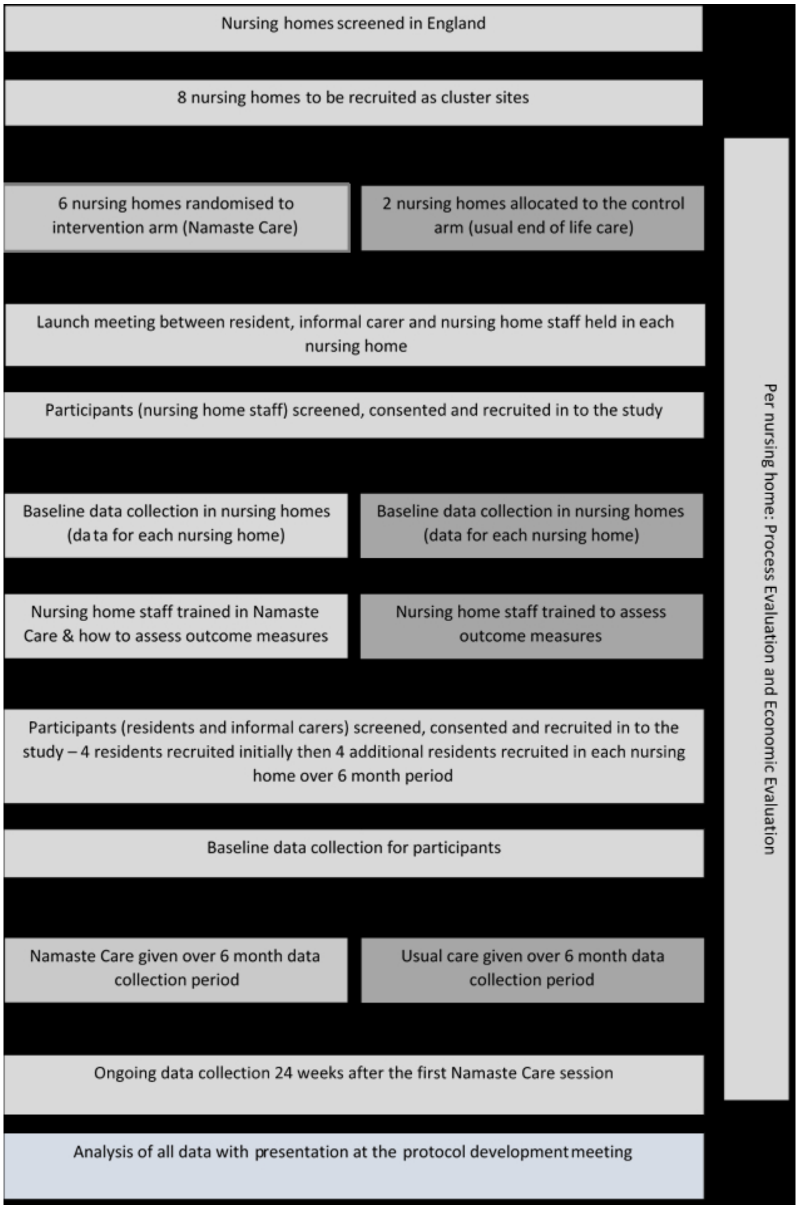

Flow diagram outlining the process of the study

SPIRIT checklist for Namaste Trial Protocol Paper

Section/item	Item No	Description	On Page No:
**Administrative information**			
Title	1	Descriptive title identifying the study design, population, interventions, and, if applicable, trial acronym	1/3
Trial registration	2a	Trial identifier and registry name. If not yet registered, name of intended registry	2/28
	2b	All items from the World Health Organization Trial Registration Data Set	N/A
Protocol version	3	Date and version identifier	17/1
Funding	4	Sources and types of financial, material, and other support	26
Roles and responsibilities	5a	Names, affiliations, and roles of protocol contributors	1
	5b	Name and contact information for the trial sponsor	8/18
	5c	Role of study sponsor and funders, if any, in study design; collection, management, analysis, and interpretation of data; writing of the report; and the decision to submit the report for publication, including whether they will have ultimate authority over any of these activities	25/6
	5d	Composition, roles, and responsibilities of the coordinating centre, steering committee, endpoint adjudication committee, data management team, and other individuals or groups overseeing the trial, if applicable (see Item 21a for data monitoring committee)	15/8-20

For peer review only

**Introduction**			
Background and rationale	6a	Description of research question and justification for undertaking the trial, including summary of relevant studies (published and unpublished) examining benefits and harms for each intervention	4/3 – 5/4
	6b	Explanation for choice of comparators	N/A
Objectives	7	Specific objectives or hypotheses	5/32-6/14
Trial design	8	Description of trial design including type of trial (eg, parallel group, crossover, factorial, single group), allocation ratio, and framework (eg, superiority, equivalence, noninferiority, exploratory)	6/17-21
**Methods: Participants, interventions, and outcomes**			
Study setting	9	Description of study settings (eg, community clinic, academic hospital) and list of countries where data will be collected. Reference to where list of study sites can be obtained	6/25
Eligibility criteria	10	Inclusion and exclusion criteria for participants. If applicable, eligibility criteria for study centres and individuals who will perform the interventions (eg, surgeons, psychotherapists)	6/28 – 8/8
Interventions	11a	Interventions for each group with sufficient detail to allow replication, including how and when they will be administered	9/29 -10/26
	11b	Criteria for discontinuing or modifying allocated interventions for a given trial participant (eg, drug dose change in response to harms, participant request, or improving/worsening disease)	9/10-12

For peer review only - http://bmjopen.bmj.com/site/about/guidelines.xhtml

	11c	Strategies to improve adherence to intervention protocols, and any procedures for monitoring adherence (eg, drug tablet return, laboratory tests)	10/17-21
	11d	Relevant concomitant care and interventions that are permitted or prohibited during the trial	N/A
Outcomes	12	Primary, secondary, and other outcomes, including the specific measurement variable (eg, systolic blood pressure), analysis metric (eg, change from baseline, final value, time to event), method of aggregation (eg, median, proportion), and time point for each outcome. Explanation of the clinical relevance of chosen efficacy and harm outcomes is strongly recommended	11/2-14
Participant timeline	13	Time schedule of enrolment, interventions (including any run-ins and washouts), assessments, and visits for participants. A schematic diagram is highly recommended (see Figure)	pp17-20
Sample size	14	Estimated number of participants needed to achieve study objectives and how it was determined, including clinical and statistical assumptions supporting any sample size calculations	8/10-19
Recruitment	15	Strategies for achieving adequate participant enrolment to reach target sample size	9/4-23
**Methods: Assignment of interventions (for controlled trials)**			
Allocation:			

Sequence generation	16a	Method of generating the allocation sequence (eg, computer-generated random numbers), and list of any factors for stratification. To reduce predictability of a random sequence, details of any planned restriction (eg, blocking) should be provided in a separate document that is unavailable to those who enrol participants or assign interventions	8/22-23
Allocation concealment mechanism	16b	Mechanism of implementing the allocation sequence (eg, central telephone; sequentially numbered, opaque, sealed envelopes), describing any steps to conceal the sequence until interventions are assigned	8/22-23
Implementation	16c	Who will generate the allocation sequence, who will enrol participants, and who will assign participants to interventions	8/22-23
Blinding (masking)	17a	Who will be blinded after assignment to interventions (eg, trial participants, care providers, outcome assessors, data analysts), and how	8/26-31
	17b	If blinded, circumstances under which unblinding is permissible, and procedure for revealing a participant's allocated intervention during the trial	N/A
**Methods: Data collection, management, and analysis**			
Data collection methods	18a	Plans for assessment and collection of outcome, baseline, and other trial data, including any related processes to promote data quality (eg, duplicate measurements, training of assessors) and a description of study instruments (eg, questionnaires, laboratory tests) along with their reliability and validity, if known. Reference to where data collection forms can be found, if not in the protocol	11/17 -12/30

	18b	Plans to promote participant retention and complete follow-up, including list of any outcome data to be collected for participants who discontinue or deviate from intervention protocols	9/19-23
Data management	19	Plans for data entry, coding, security, and storage, including any related processes to promote data quality (eg, double data entry; range checks for data values). Reference to where details of data management procedures can be found, if not in the protocol	15/20-21
Statistical methods	20a	Statistical methods for analysing primary and secondary outcomes. Reference to where other details of the statistical analysis plan can be found, if not in the protocol	13/14 – 14/19
	20b	Methods for any additional analyses (eg, subgroup and adjusted analyses)	N/A
	20c	Definition of analysis population relating to protocol non-adherence (eg, as randomised analysis), and any statistical methods to handle missing data (eg, multiple imputation)	13/2-11
**Methods: Monitoring**			
Data monitoring	21a	Composition of data monitoring committee (DMC); summary of its role and reporting structure; statement of whether it is independent from the sponsor and competing interests; and reference to where further details about its charter can be found, if not in the protocol. Alternatively, an explanation of why a DMC is not needed	15/12-18

	21b	Description of any interim analyses and stopping guidelines, including who will have access to these interim results and make the final decision to terminate the trial	13/2-11
Harms	22	Plans for collecting, assessing, reporting, and managing solicited and spontaneously reported adverse events and other unintended effects of trial interventions or trial conduct	15/2-7
Auditing	23	Frequency and procedures for auditing trial conduct, if any, and whether the process will be independent from investigators and the sponsor	15/12-18
**Ethics and dissemination**			
Research ethics approval	24	Plans for seeking research ethics committee/institutional review board (REC/IRB) approval	16/1
Protocol amendments	25	Plans for communicating important protocol modifications (eg, changes to eligibility criteria, outcomes, analyses) to relevant parties (eg, investigators, REC/IRBs, trial participants, trial registries, journals, regulators)	16/1
Consent or assent	26a	Who will obtain informed consent or assent from potential trial participants or authorised surrogates, and how (see Item 32)	9/2-26
	26b	Additional consent provisions for collection and use of participant data and biological specimens in ancillary studies, if applicable	N/A
Confidentiality	27	How personal information about potential and enrolled participants will be collected, shared, and maintained in order to protect confidentiality before, during, and after the trial	13/2-3

For peer review only

Declaration of interests	28	Financial and other competing interests for principal investigators for the overall trial and each study site	25/6-9
Access to data	29	Statement of who will have access to the final trial dataset, and disclosure of contractual agreements that limit such access for investigators	15/19
Ancillary and post-trial care	30	Provisions, if any, for ancillary and post-trial care, and for compensation to those who suffer harm from trial participation	N/A
Dissemination policy	31a	Plans for investigators and sponsor to communicate trial results to participants, healthcare professionals, the public, and other relevant groups (eg, via publication, reporting in results databases, or other data sharing arrangements), including any publication restrictions	16/6-9
	31b	Authorship eligibility guidelines and any intended use of professional writers	25/2-5
	31c	Plans, if any, for granting public access to the full protocol, participant-level dataset, and statistical code	N/A
**Appendices**			
Informed consent materials	32	Model consent form and other related documentation given to participants and authorised surrogates	N/A
Biological specimens	33	Plans for collection, laboratory evaluation, and storage of biological specimens for genetic or molecular analysis in the current trial and for future use in ancillary studies, if applicable	N/A

*It is strongly recommended that this checklist be read in conjunction with the SPIRIT 2013 Explanation & Elaboration for important clarification on the items. Amendments to the protocol should be tracked and dated. The SPIRIT checklist is copyrighted by the SPIRIT

For peer review only
