# Supplementary material for: Namaste Care in nursing care homes for people with advanced dementia: protocol for a feasibility randomised controlled trial
Source: BMJ Open. 2018 Nov 25;8(11):e026531. doi: 10.1136/bmjopen-2018-026531 (PMC6254402; doi:10.1136/bmjopen-2018-026531)
Supplement: Reviewer comments [file bmjopen-2018-026531.reviewer_comments.pdf]

## PEER REVIEW HISTORY

BMJ Open publishes all reviews undertaken for accepted manuscripts. Reviewers are asked to complete a checklist review form (**http://bmjopen.bmj.com/site/about/resources/checklist.pdf**) and are provided with free text boxes to elaborate on their assessment. These free text comments are reproduced below.

## ARTICLE DETAILS

| | |
|---|---|
| **TITLE (PROVISIONAL)** | Namaste Care in nursing care homes with people with advanced dementia: protocol for a feasibility randomised controlled trial |
| **AUTHORS** | Froggatt, Katherine; Patel, Shakil; Perez Algorta, Guillermo; Bunn, Frances; Burnside, Girvan; Coast, Joanna; Dunleavy, Lesley; Goodman, Claire; Hardwick, Ben; Kinley, Julie; Preston, Nancy J.; Walshe, Catherine |

This article was not externally reviewed at BMJ Open. Protocols that have been independently assessed prior to submission to BMJ Open are usually fast-tracked to publication on the grounds that further substantial changes will not be possible. This independent assessment will usually be external, independent review for both a substantial grant award from a non-commercial or government funder and ethics approval.
